# 14-3-3ζ coordinates adipogenesis of visceral fat

**DOI:** 10.1038/ncomms8671

**Published:** 2015-07-29

**Authors:** Gareth E. Lim, Tobias Albrecht, Micah Piske, Karnjit Sarai, Jason T. C Lee, Hayley S. Ramshaw, Sunita Sinha, Mark A. Guthridge, Amparo Acker-Palmer, Angel F. Lopez, Susanne M. Clee, Corey Nislow, James D. Johnson

**Affiliations:** 1 Department of Cellular and Physiological Sciences, University of British Columbia, Vancouver, British Columbia, Canada V6T 1Z3; 2 The Centre for Cancer Biology, SAPathology and University of South Australia, Adelaide, SA 5000, Australia; 3 Department of Pharmaceutical Sciences, University of British Columbia, Vancouver, British Columbia, Canada V6T 1Z3; 4 Division of Blood Cancers, Australian Centre for Blood Diseases, Monash University, Melbourne, Victoria, VIC 3004, Australia; 5 Institute of Cell Biology and Neuroscience and Buchmann Institute for Molecular Life Sciences, University of Frankfurt, Frankfurt am Main 60438, Germany

## Abstract

The proteins that coordinate complex adipogenic transcriptional networks are poorly understood. 14-3-3ζ is a molecular adaptor protein that regulates insulin signalling and transcription factor networks. Here we report that 14-3-3ζ-knockout mice are strikingly lean from birth with specific reductions in visceral fat depots. Conversely, transgenic 14-3-3ζ overexpression potentiates obesity, without exacerbating metabolic complications. Only the 14-3-3ζ isoform is essential for adipogenesis based on isoform-specific RNAi. Mechanistic studies show that 14-3-3ζ depletion promotes autophagy-dependent degradation of C/EBP-δ, preventing induction of the master adipogenic factors, Pparγ and C/EBP-α. Transcriptomic data indicate that 14-3-3ζ acts upstream of hedgehog signalling-dependent upregulation of *Cdkn1b/*p27^Kip1^. Indeed, concomitant knockdown of p27^Kip1^ or Gli3 rescues the early block in adipogenesis induced by 14-3-3ζ knockdown *in vitro*. Adipocyte precursors in 14-3-3ζKO embryos also appear to have greater Gli3 and p27^Kip1^ abundance. Together, our *in vivo* and *in vitro* findings demonstrate that 14-3-3ζ is a critical upstream driver of adipogenesis.

## Abstract (web summary)

14-3-3 family proteins are adaptor proteins involved in various cellular functions. Here Lim *et al*. show that 14-3-3ζ regulates adipogenesis *in vitro*, and the formation of visceral fat in mice, by reducing autophagic degradation of the adipogenic master transcription factor C/EBP-δ.

## Graphical abstract

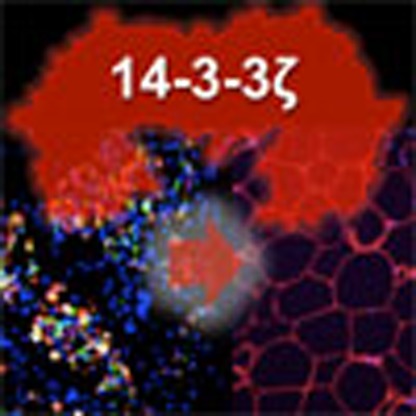

Obesity, a risk factor for many diseases, can result from increased proliferation and/or differentiation of adipocyte precursor cells^1^,^2^,^3^. Modulating processes that control the expansion or differentiation of adipose tissue may yield promising drug targets^4^,^5^, but an incomplete understanding of the complex gene networks that underlie adipogenesis stands in the way of this goal. Precise temporal and spatial control of specific protein–protein and protein–DNA interactions drive the induction of master adipogenic factors^6^,^7^. The principle events underlying adipogenesis involve the nuclear translocation of CCAAT/enhancer-binding protein (C/EBP)-β and C/EBP-δ to initiate the adipogenic programme, leading to the expression of C/EBP-α and peroxisome proliferator-activated receptor-γ (Pparγ) during terminal adipocyte differentiation^6^,^7^. Currently, it is not known which proteins ensure the accurate binding and localization of such transcriptional complexes in adipocytes.

Signalling events and networks can be coordinated by adaptor proteins, which facilitate the proper localization of effector molecules, transcription factors and kinases^8^,^9^,^10^. Adaptor proteins, such as those of the highly conserved 14-3-3 protein family, remain poorly understood compared with other classes of signalling molecules. These adapters interact with transcription factors harbouring canonical phosphorylated serine and threonine motifs, facilitating their nuclear import or export^9^,^10^,^11^. Little is known about the specific adaptor proteins that coordinate the stability and/or nuclear translocation of critical adipogenic factors, but 14-3-3 proteins are ideal candidates. While 14-3-3 isoforms across species display a high degree of homology and may have some functional redundancy, each isoform could perform unique, context-specific functions^12^,^13^. We have demonstrated that not all 14-3-3 isoforms have equal roles in pancreatic β-cell survival^14^. Whether 14-3-3 isoforms specifically regulate other physiological processes, such as adipogenesis, is still unclear due to the lack of functional studies employing side-by-side comparisons.

Aberrant 14-3-3 protein abundance has been proposed to drive the development of various chronic diseases^15^,^16^. In fact, elevations in 14-3-3β, 14-3-3γ and 14-3-3ζ protein levels have been reported in adipose tissue from obese individuals^17^,^18^,^19^. Whether such increases have causal roles in the development of obesity is unclear, but these observations suggest pro-obesogenic roles of this family of adaptor proteins. Given the ability of 14-3-3 proteins to control differentiation in other cell types^20^, it is reasonable to hypothesize that one or more 14-3-3 proteins could play pivotal roles in adipogenesis.

We report herein that out of the seven 14-3-3 isoforms, only 14-3-3ζ plays an essential role in adipocyte differentiation *in vitro*. Deletion of 14-3-3ζ in mice causes marked reductions in adipose tissue within specific depots, as well as metabolic impairments, while 14-3-3ζ overexpression promotes fat tissue expansion without deleterious metabolic defects. Targeted analysis and unbiased transcriptomics reveals complex mechanisms whereby 14-3-3ζ regulates a diverse set of parallel and sequential events to drive the adipogenic programme. Loss of 14-3-3ζ causes the aberrant expression of hedgehog signalling effector, Gli3, and the cyclin-dependent kinase inhibitor, p27^Kip1^, which attenuates adipogenesis. Taken together, our data support the concept that 14-3-3ζ is a critical upstream regulator of adipocyte differentiation. Therefore, targeting 14-3-3ζ and components of its interactome may represent novel therapeutic targets for obesity.

## Results

### 14-3-3ζ regulates adiposity and adipocyte differentiation

**Figure 1:**
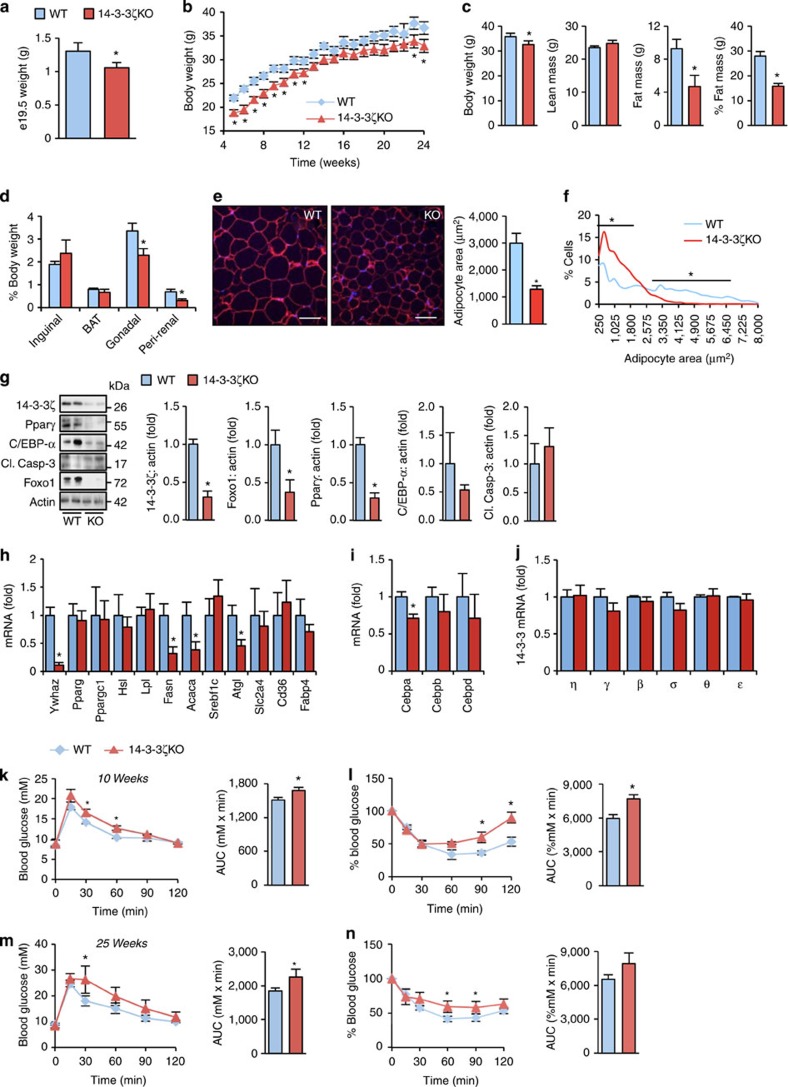
Reduced adiposity and adipocyte maturity in mice lacking 14-3-3ζ. (**a**) Weights of e19.5 wild-type (WT) and 14-3-3ζ knockout (14-3-3ζKO) (*n*=3 per group). (**b**) Body weights of 14-3-3ζKO mice and littermate control wild-type (WT) mice (*n*=10–12 per group). (**c**) DEXA body composition analysis of WT and 14-3-3ζKO at 24 weeks of age (*n*=5–7 per group). (**d**) Weights of inguinal, brown adipose (BAT), gonadal and peri-renal fat pads relative to total body weight of male WT and 14-3-3ζKO mice (*n*=4 per genotype). (**e**,**f**) Analysis of white adipocyte area (**e**) and size distribution (**f**) of inguinal fat tissue, as assessed by perilipin staining, from 26 week old WT and KO mice (*n*=3 per genotype). (**g**–**i**) Immunoblot (**g**) and quantitative PCR measurements (**h**,**i**) of pro-adipogenic factors or mature adipocyte markers, respectively, from gonadal fat pads (*n*=4–6 per group). (**j**) Comparison of remaining 14-3-3 isoforms in gonadal white adipose tissue from WT and 14-3-3ζKO mice (*n*=5 per group). (**k**–**n**) Intraperitoneal glucose (**k**,**m**; 2 g kg^−1^ b.w.) and insulin (**l**,**n**; 1.5 U kg^−1^) tolerance tests on WT and 14-3-3ζKO littermates at 10 (**k**,**l**) and 25 (**m**,**n**) weeks of age. Area-under-the-curve measurements are shown (*n*=7–8 mice per group). Error bars represent s.e.m. Significant differences between WT and KO mice are indicated by *P<0.05 (assessed by Student's *t*-test).

To understand the developmental and physiological roles of 14-3-3ζ, we examined 14-3-3ζ-knockout mice (14-3-3ζKO). This previously generated mouse model had been used to implicate 14-3-3ζ in PI3K activation^9^, but characterizations of body composition and/or energy homeostasis had not been reported. Before birth, 14-3-3ζKO embryos were smaller and weighed significantly less than wild-type embryos (Fig. 1a; Supplementary Fig. 1a). Despite catching up in length to wild-type mice in early adulthood (24 weeks), 14-3-3ζKO mice were significantly lighter than wild-type controls due to significantly reduced fat mass as revealed by DEXA body composition analysis (Fig. 1b,c; Supplementary Fig. 1b,c). Analysis of subcutaneous and visceral fat depots showed significant decreases in both gonadal fat and peri-renal fat, but no effect on inguinal fat or brown adipose tissue (Fig. 1d; Supplementary Fig. 1d). The reduction in fat mass was reflected by significantly reduced fasting and random-fed plasma leptin concentrations, as well as significantly lower triglyceride levels in 14-3-3ζKO mice (Supplementary Fig. 1e). The decrease in adiposity was not associated with alterations in energy expenditure or food intake (Supplementary Fig. 1f), suggestive of a specific function of 14-3-3ζ in adipocytes.

Analysis of white adipocyte morphology in gonadal fat pad cross-sections showed significantly smaller adipocytes in 14-3-3ζKO mice (Fig. 1e,f), suggesting a less mature cellular phenotype. Protein abundance of Foxo1 and Pparγ, markers indicative of mature adipocytes, were significantly reduced in 14-3-3ζKO gonadal white adipocytes (Fig. 1g). *Pparg* mRNA was not altered, suggesting post-transcriptional regulation of this master adipogenesis regulator (Fig. 1h). Of the C/EBP isoforms, only mRNA expression of C/EBP-α was significantly reduced in 14-3-3ζKO adipocytes (Fig. 1i), as were *Fasn* and *Atgl* mRNA (Fig. 1h). These observations suggest that 14-3-3ζ deletion results in poorly differentiated, immature adipocytes. The decrease in fat pad size was not associated with increased steady-state apoptosis, quantified by western blot analysis of cleaved caspase-3 (Fig. 1g). Quantitative PCR confirmed that expression levels of remaining 14-3-3 isoforms were unchanged in 14-3-3ζKO mice (Fig. 1j), which indicates that any effects in adiposity were specific to changes in 14-3-3ζ expression and not influenced by alterations in the expression of other 14-3-3 members. Thus, 14-3-3ζ controls the development and maturity of adipocytes *in vivo*.

The lean phenotype of the 14-3-3ζKO mice prompted us to examine whether excess lipids had accumulated in non-adipose tissue sites. Indeed, 14-3-3ζKO livers exhibited mild steatosis (Supplementary Fig. 1g). Hepatic *Hsl* expression was decreased in 14-3-3ζKO mice (Supplementary Fig. 1h), but no differences in genes encoding gluconeogenic enzymes were observed between wild-type or knockout mice (Supplementary Fig. 1i).

### Decreased glucose and insulin tolerance in 14-3-3ζKO mice

Decreased 14-3-3ζ abundance is associated with insulin resistance in humans^21^, but it is unclear if this relationship is causative. Thus, we evaluated glucose homeostasis and insulin sensitivity in 14-3-3ζKO mice. No differences in fasting glucose levels were observed between groups (Fig. 1k,m). Intraperitoneal glucose and insulin tolerance tests revealed that 14-3-3ζKO mice were mildly glucose intolerant and exhibited mild systemic insulin resistance (Fig. 1k–n). Significantly higher fasting plasma insulin concentrations were seen in 14-3-3ζKO mice (Supplementary Table 1), but the decrease in insulin sensitivity was not due to differences in circulating adiponectin concentrations in 14-3-3ζKO mice (Supplementary Table 1). The decrease in insulin sensitivity was associated with decreased Akt activation in livers of 14-3-3ζKO mice following an intraperitoneal insulin bolus (Supplementary Fig. 1j).

### Over-expression of 14-3-3ζ promotes healthy fat expansion

**Figure 2:**
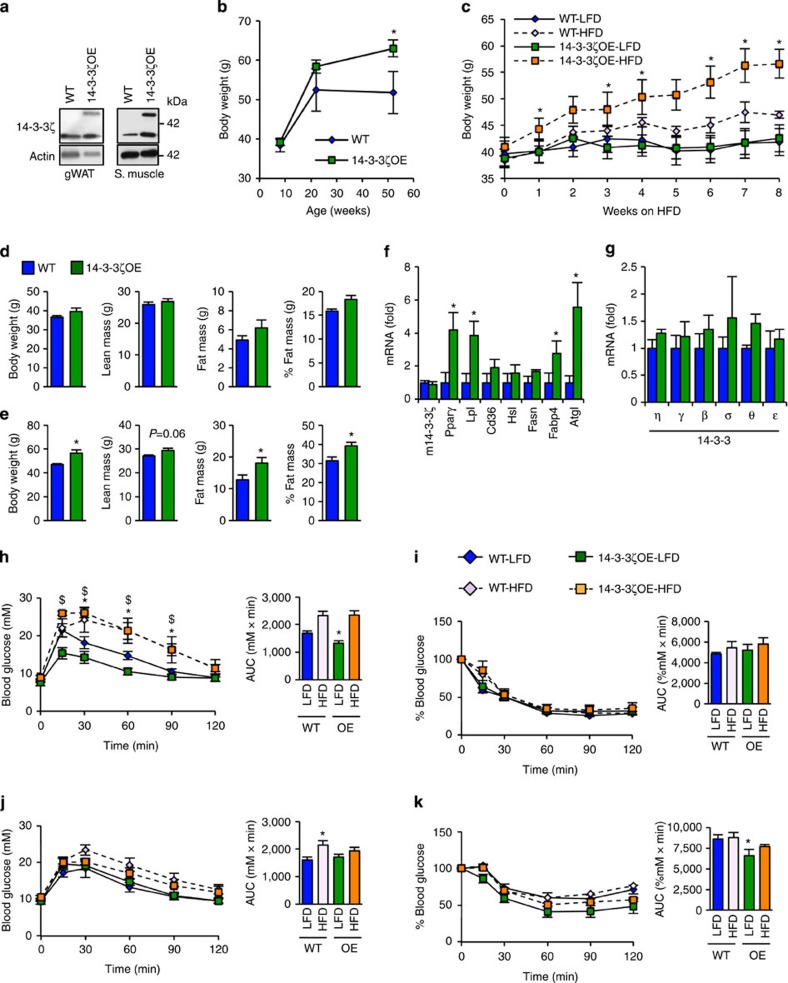
Overexpression of 14-3-3ζ is sufficient for age-associated and high-fat diet-associated weight gain. (**a**) Expression of the TAP-tagged 14-3-3ζ (50 kDa) and endogenous 14-3-3ζ (30 kDa) in gonadal white adipose tissue (gWAT) and skeletal muscle (*n*=3 per group). (**b**) Body weights of WT and 14-3-3ζ-over-expressing transgenic (14-3-3ζOE) mice were measured for 1 year (*n*=4–9 per group; *P<0.05, assessed by Student's *t*-test). (**c**) Weekly body weights of 12 week old WT and 14-3-3ζOE mice fed a high-fat diet (HFD, 60% fat) or the corresponding control diet (LFD, 10% fat) for 8 weeks (*n*=6–8 per group, *P<0.05 when comparing HFD-WT to HFD-14-3-3ζOE mice, assessed by Student's *t*-test). (**d**,**e**) WT or 14-3-3ζOE mice were subject to DEXA body composition analysis before exposure to high-fat diet (**d**) or after 8 weeks (**e**) (*n*=6–8 per group, *P<0.05, assessed by Student's *t*-test). (**f**) Quantitative PCR measurements of mature white adipocyte markers from WT and 14-3-3ζOE mice (*n*=6–8 per group; *P<0.05, assessed by Student's *t*-test). (**g**) Expression profile of remaining 14-3-3 isoforms in inguinal white adipose tissue from WT and 14-3-3ζOE mice (*n*=5 per group). (**h**,**i**) Glucose tolerance (2 g kg^−1^ b.w.; **h**) and insulin tolerance (1.5 U kg^−1^ b.w.; **i**) tests were administered to WT or 14-3-3ζOE mice after 8 weeks of high-fat diet exposure (*n*=6–8 per group, *P<0.05 when comparing WT-LFD with WT-HFD, $*P*<0.05 when comparing 14-3-3ζOE-LFD with 14-3-3ζOE-HFD, assessed by one-way ANOVA). (**j**,**k**) Glucose tolerance (**j**) and insulin tolerance (0.75 U kg^−1^ b.w.; **k**) tests were administered to WT or 14-3-3ζOE mice after 2 weeks of high-fat diet exposure (*n*=5–9 per group; *P<0.05, assessed by two-way ANOVA when comparing WT-LFD with 14-3-3ζOE–LFD). Error bars represent s.e.m.

Obesity has been associated with increased adipose tissue 14-3-3 protein abundance in several studies^17^,^18^,^19^, although it is unknown whether this change alone is sufficient to increase adiposity. To test this hypothesis, we studied transgenic mice with modest global overexpression of 14-3-3ζ under the control of the ubiquitin-C promoter^22^. Levels of the transgene were equally expressed in insulin-sensitive gonadal white adipose tissue and skeletal muscle (Fig. 2a), as well as other tissues^22^. At 52 weeks of age, 14-3-3ζ transgenic mice were significantly heavier than their wild-type littermate controls even when fed a normal chow diet (Fig. 2b). Notably, 14-3-3ζ transgenic mice did not develop glucose intolerance or insulin resistance (Supplementary Fig. 2a–d), suggesting expansion of metabolically neutral adipose tissue.

Next, we tested whether overexpression of 14-3-3ζ promotes increased capacity for adipose expansion in the context of nutrient excess. Indeed, high-fat diet feeding for 8 weeks triggered significantly greater weight gain and fat mass in 14-3-3ζ overexpressing mice when compared with wild-type littermates (Fig. 2c–e). Furthermore, these mice had significantly higher *Pparγ*, *Lpl* and *Ap2* expression in gonadal white adipocytes (Fig. 2f). Overexpression of 14-3-3ζ did not affect expression of other 14-3-3 isoforms in adipose tissue (Fig. 2g), suggesting that these effects are solely due to increased levels of 14-3-3ζ. High-fat diet promoted the expected glucose intolerance in both wild-type and 14-3-3ζ-overexpressing mice (Fig. 2h). However, despite markedly greater weight gain, there were no additional deleterious effects on glucose homeostasis in 14-3-3ζ-overexpressing mice. Similarly, no additional negative effects on insulin sensitivity (1.5 U kg^−1^) resulted from the increased fat mass in 14-3-3ζ-overexpressing animals (Fig. 2i). Using a lower dose of insulin (0.75 U kg^−1^), we found that 14-3-3ζ-overexpressing mice were actually more insulin sensitive than wild-type littermate controls (Fig. 2k). The degree of hepatic steatosis following high-fat diet exposure was similar between groups, and no differences in circulating plasma free fatty acids and triglycerides were detected (Supplementary Fig. 2e–g). Analysis of genes associated with hepatic lipid metabolism and gluconeogenesis revealed that 14-3-3ζ-overexpressing mice had decreased transcript abundance of *Fasn*, *Srebp*-1c and *Acc* (Supplementary Fig. 2h,i). Collectively, our findings suggest that 14-3-3ζ is necessary and sufficient to control obesity *in vivo*.

### 14-3-3ζ is required for adipocyte differentiation *in vitro*

**Figure 3:**
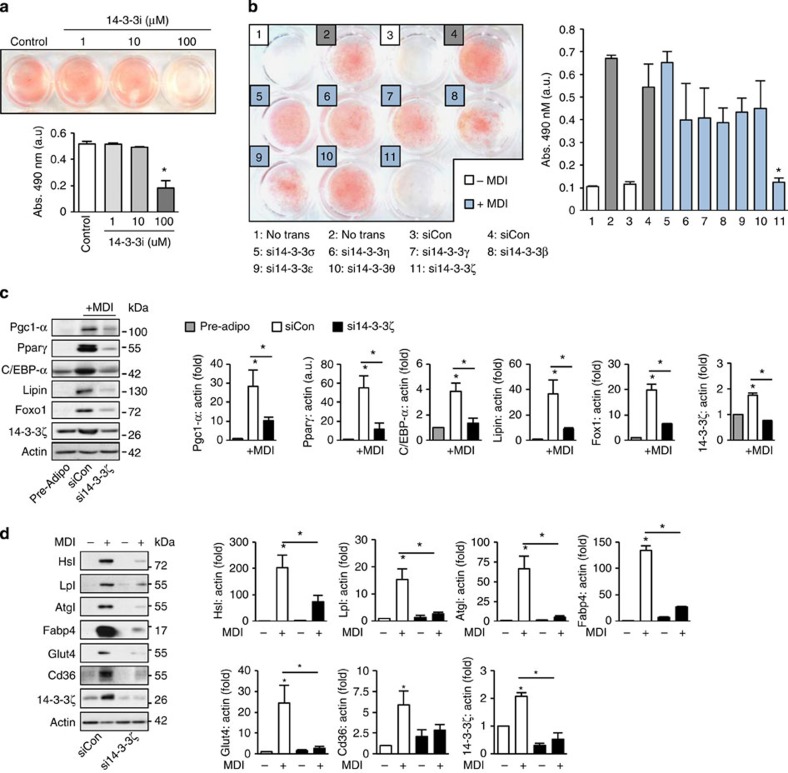
siRNA-mediated knockdown of all 14-3-3 proteins reveals the 14-3-3ζ as the critical regulator of adipocyte differentiation. (**a**) 3T3-L1 pre-adipocytes were pretreated with a cell-permeable pan-14-3-3 inhibitor (14-3-3i) and differentiated by MDI. Differentiation was visualized by Oil-Red-O incorporation (*n*=4 independent experiments, *P<0.05, assessed by one-way ANOVA). (**b**) 3T3-L1 pre-adipocytes were transfected with 14-3-3 isoform-specific siRNA followed by differentiation with MDI. Differentiation was measured by Oil Red-O incorporation (*n*=4 independent experiments, *P<0.05, assessed by Student's *t*-test). (**c**,**d**) Pro-adipogenic factors (**c**) or mature adipocyte markers (**d**) were measured 48 h or 7 days, respectively, after differentiation in control- (siCon) and si14-3-3ζ-transfected 3T3-L1 cells (*n*=4 per group, *P<0.05, assessed by one- or two-way ANOVA). Error bars represent s.e.m.

The *in vivo* studies described above define critical roles of 14-3-3ζ in obesity. To define the isoform specificity of these effects and the mechanisms by which 14-3-3ζ facilitates adipocyte differentiation, we utilized three *in vitro* models. We first used 3T3-L1 pre-adipocytes, which recapitulate many signalling and transcriptional events leading to the maturation of primary adipocytes^23^, and pre-treated cells with a pan-14-3-3 small molecule inhibitor that disrupts the interaction of 14-3-3 proteins with their target proteins^24^. Inhibition of 14-3-3 proteins blocked adipocyte differentiation and demonstrated a requirement for at least one member of this molecular adaptor family (Fig. 3a). Quantitative PCR revealed changes in expression levels of several 14-3-3 isoforms during *in vitro* adipogenesis, but only 14-3-3ζ mRNA increased and stayed increased during the initial and critical 48- hour period (Supplementary Fig. 3a). To test the individual requirement of each isoform in adipogenesis, validated isoform-specific siRNAs^14^ were transfected into 3T3-L1 pre-adipocytes before differentiation (Supplementary Fig. 3b,c). 14-3-3ζ was the only isoform required for differentiation, as assessed by Oil Red-O staining (Fig. 3b). Knockdown of 14-3-3ζ had no effect on the expression of remaining isoforms (Supplementary Fig. 3c). The effect of 14-3-3ζ depletion was not due to a delay in differentiation, as si14-3-3ζ-transfected 3T3-L1 cells incubated for up to 14 days still did not undergo adipogenesis (Supplementary Fig. 3d). 14-3-3ζ knockdown had similar inhibitory effects on adipogenesis in 3T3-F442A cells (Supplementary Fig. 4a)^25^. The prevention of differentiation was not due to off-target effects of the RNAi approach because embryonic fibroblasts derived from 14-3-3ζKO mice also failed to fully differentiate into adipocytes (Supplementary Fig. 4c). Collectively, our data from three independent, *in vitro* 14-3-3ζ loss-of-function models clearly suggest an essential, cell autonomous role for 14-3-3ζ, but not other 14-3-3 family members, in the process of adipocyte differentiation.

We next assessed the molecular consequences of 14-3-3ζ knockdown *in vitro*. Measurement of early-stage adipogenic transcription factors and mature adipocyte markers revealed a critical role for 14-3-3ζ is this gene network (Fig. 3c,d). Specifically, 14-3-3ζ knockdown prevented the increased abundance of C/EBP-α, Pparγ and Foxo1 (Fig. 3c), which are essential master transcriptional regulators of adipogenesis^7^. The decreases in Foxo1 and Pparγ protein abundance in 14-3-3ζ-deficient 3T3-L1 cells recapitulated the significantly reduced expression of these master adipogenic factors in 14-3-3ζKO adipocytes *in vivo* (Fig. 1h). Depletion of 14-3-3ζ also prevented the induction of adipocyte-specific proteins associated with lipid metabolism (Fig. 3d). In 3T3-F442A cells, the defects in adipogenesis caused by depletion of 14-3-3ζ were also associated with impaired expression of various mature adipocyte markers (Supplementary Fig. 4b). Together, with the *in vivo* data, these results further support a key upstream role of 14-3-3ζ in adipogenesis.

**Figure 4:**
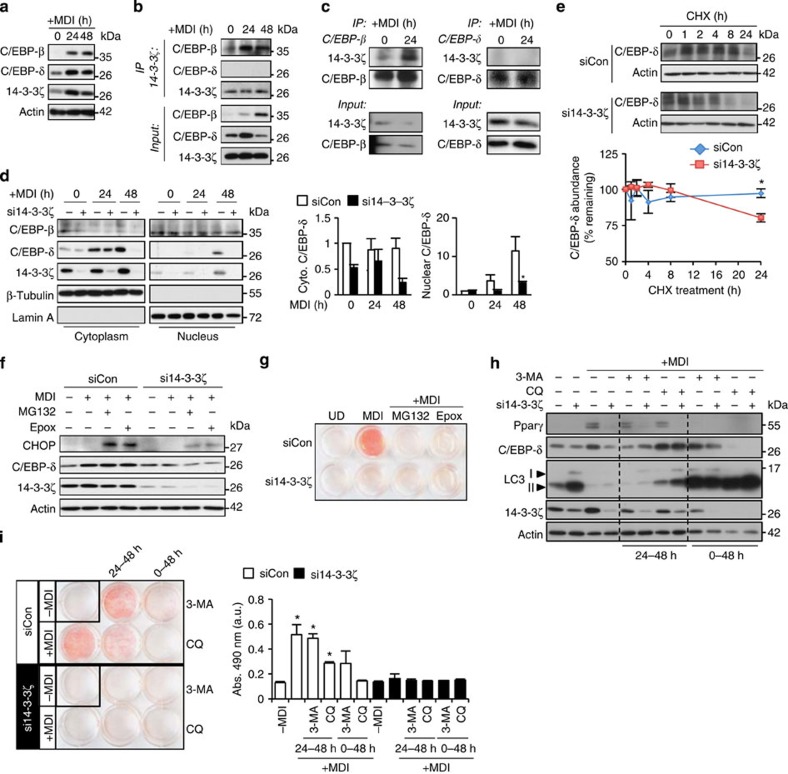
Loss of 14-3-3ζ leads to rapid, autophagy-dependent degradation of C/EBP-δ. (**a**) C/EBP-β and C/EBP-δ protein abundance during the first 48 h of differentiation in 3T3-L1 cells were measured by immunoblotting (*n*=3 biological replicates). (**b**,**c**) Co-immunoprecipitation of endogenous (**b**) 14-3-3ζ or (**c**) C/EBP-β or -δ from lysates of 3T3-L1 cells exposed to MDI for 0, 24 or 48 h (*n*=4 independent experiments). (**d**) Nuclear and cytoplasmic fractions were obtained from si14-3-3ζ-transfected 3T3-L1 adipocytes to examine the subcellular localization and abundance of C/EBP-β and C/EBP-δ during differentiation (*n*=4 independent experiments). (**e**) Inhibition of protein translation with 10 μM cycloheximide (CHX) was used to examine whether loss of C/EBP-δ following knockdown of 14-3-3ζ was due to degradation or effects on protein translation (*n*=4 per group, *P<0.05, assessed by Student's *t*-test). (**f**) Immunblotting was used to examine whether proteasomal inhibition by 10 μM MG132 or 100 μM epoxomicin (Epox) could increase the abundance of C/EBP-δ or the closely related protein, CHOP (*n*=3 independent experiments). (**g**) 10 μM MG132 or 100 μM Epox was used to examine whether proteasomal inhibition could restore si14-3-3ζ-mediated inhibition of differentiation (*n*=3 independent experiments). (**h**) 3T3-L1 pre-adipocytes were induced with MDI and treated with 3-methyladenine (3-MA) and chloroquine (CQ) for 48 h or the last 24 h (24–48 h) of the induction period. Inhibition of autophagy was verified by examining the accumulation of LC3 (*n*=4 independent experiments). (**i**) 14-3-3ζ-depleted 3T3-L1 cells were incubated with 3-methyladenine or chloroquine for 48 h or the last 24 h during induction of differentiation by MDI. Cells were stained with Oil Red-O to assess adipocyte differentiation (*n*=3 independent experiments; *P<0.05, assessed by ANOVA). Error bars represent s.e.m.

To further elucidate the molecular mechanisms involved in controlling this master adipogenic network, we tested the possibility that 14-3-3ζ might control the abundance of C/EBP-β and C/EBP-δ, regulators of transcription during the early stages of adipogenesis^7^. During the critical first 48 h of 3T3-L1 cell differentiation, we observed parallel increases in the abundance of 14-3-3ζ together with C/EBP-β and C/EBP-δ (Fig. 4a). Pull-down experiments showed that C/EBP-β, but not C/EBP-δ, associated with endogenous 14-3-3ζ in differentiating cells (Fig. 4b,c). As 14-3-3 proteins participate in the nuclear transport of key adipocyte transcription factors^11^, we tested whether 14-3-3ζ knockdown might affect the nuclear translocation of C/EBP-β or C/EBP-δ during differentiation. Indeed, subcellular fractionation experiments demonstrated that the amount of nuclear-localized 14-3-3ζ increased during the differentiation process (Fig. 4d). 14-3-3ζ knockdown had no impact on C/EBP-β nuclear import (Fig. 4d). However, 14-3-3ζ depletion led to an unexpected and marked degradation of C/EBP-δ during differentiation, which reduced its nuclear localization (Fig. 4d). Direct binding to 14-3-3 proteins can prevent protein degradation of target proteins^26^, but we could not detect direct association of C/EBP-δ with 14-3-3ζ (Fig. 4b), suggesting that 14-3-3ζ regulates the stability of C/EBP-δ through indirect mechanisms. We studied the effect of 14-3-3ζ depletion on C/EBP-δ degradation by treating cells with the translation inhibitor cycloheximide and confirmed that 14-3-3ζ affected the stability of C/EBP-δ (Fig. 4e). These findings place 14-3-3ζ actions at early stages of differentiation that are upstream of canonical master adipogenic transcription factors.

We next sought to determine how 14-3-3ζ controls C/EBP-δ protein stability. To examine the possibility of proteasome-mediated degradation of C/EBP-δ, we treated 3T3-L1 pre-adipocytes with MG132 or epoxomicin, during differentiation. Neither inhibitors affected C/EBP-δ stability (Fig. 4f,g) nor were they able to overcome the 14-3-3ζ siRNA-mediated inhibition of 3T3-L1 differentiation into adipocytes (Fig. 4g). Paradoxically, we also observed a 14-3-3ζ-dependent increase in the abundance of CHOP, which is known to inhibit C/EBP-β and C/EBP-δ^27^ and may account for the failure to restore adipogenesis in this context (Fig. 4f). Next we tested the hypothesis that increased autophagy accounted for the decrease in C/EBP-δ following knockdown of 14-3-3ζ, as this isoform has previously been shown to inhibit processes involved in autophagy^28^. Analysis of C/EBP-δ protein abundance revealed that inhibition of autophagy with chloroquine during the last 24 h of the induction period was able to maintain C/EBP-δ abundance in 14-3-3ζ-depleted cells. In contrast, inhibition of autophagy during the entire induction period (0–48 h) did not rescue C/EBP-δ abundance and actually promoted the loss of 14-3-3ζ (Fig. 4h). Therefore, autophagy appears to play complex, context-dependent roles in adipogenesis upstream and downstream of 14-3-3ζ. Inhibition of autophagy itself had inhibitory effects on adipocyte differentiation, and neither 3-methyladenine nor chloroquine rescued the defects in adipocyte differentiation induced by 14-3-3ζ knockdown (Fig. 4i). While these manipulations are not specific to C/EBP-δ, this observation implies that multiple, parallel 14-3-3ζ-dependent processes are important for adipocyte differentiation and prompted us to broaden the scope of our search for additional mechanisms downstream of 14-3-3ζ in the context of adipogenesis.

### 14-3-3ζ regulates cell cycle progression of pre-adipocytes

**Figure 5:**
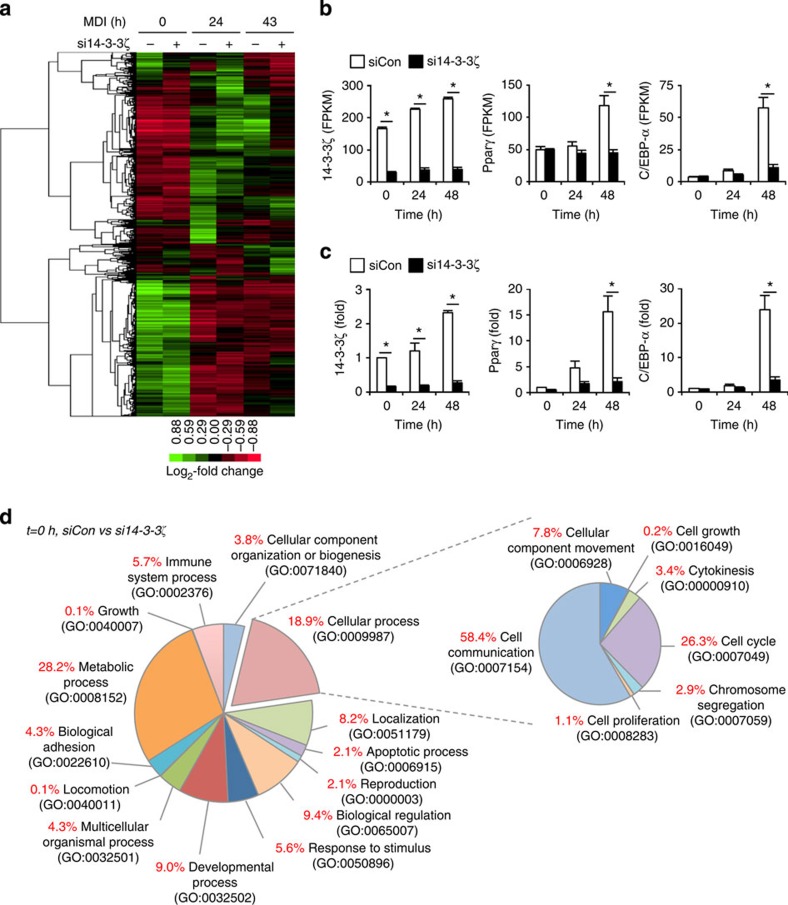
Depletion of 14-3-3ζ alters the adipogenic transcriptome. (**a**) Visualization of significantly changed genes from control or si14-3-3ζ-transfected 3T3-L1 cells at 0, 24 and 48 h of differentiation (0.05 FDR-adjusted *q*<0.05; *n*=4 per group). (**b**,**c**) Comparison of expression profiles of *14-3-3ζ*, *Pparγ* and *C/EBP-α* as determined by RNA-Seq (**b**) and quantitative PCR (**c**) (*n*=4 per group, *P<0.05, assessed by Student's *t*-test). (**d**) Panther GO analysis of differentially expressed genes between siCon- and si14-3-3ζ-transfected cells at 0 h of MDI treatment.

Loss of 14-3-3ζ may impair the nuclear import of critical transcription factors (Fig. 4d)^11^,^29^ and therefore alter the transcriptome of differentiating adipocytes. Thus, we used RNA sequencing to quantitatively measure global changes in the transcriptome and identify downstream effects of 14-3-3ζ. Over 1,200 genes were significantly altered due to induction of adipocyte differentiation or by knockdown of 14-3-3ζ (0.05 FDR-adjusted *q*<0.05) (Fig. 5a), which is not surprising given the magnitude of the phenotypic differences. Results from the transcriptomic analysis were confirmed by quantitative PCR measurement (Fig. 5b,c). Within the top 25 genes that were significantly changed, we identified genes implicated in adipogenesis, such as *Arxes* and *G0s2* (refs 30, 31) (Supplementary Table 2).

**Figure 6:**
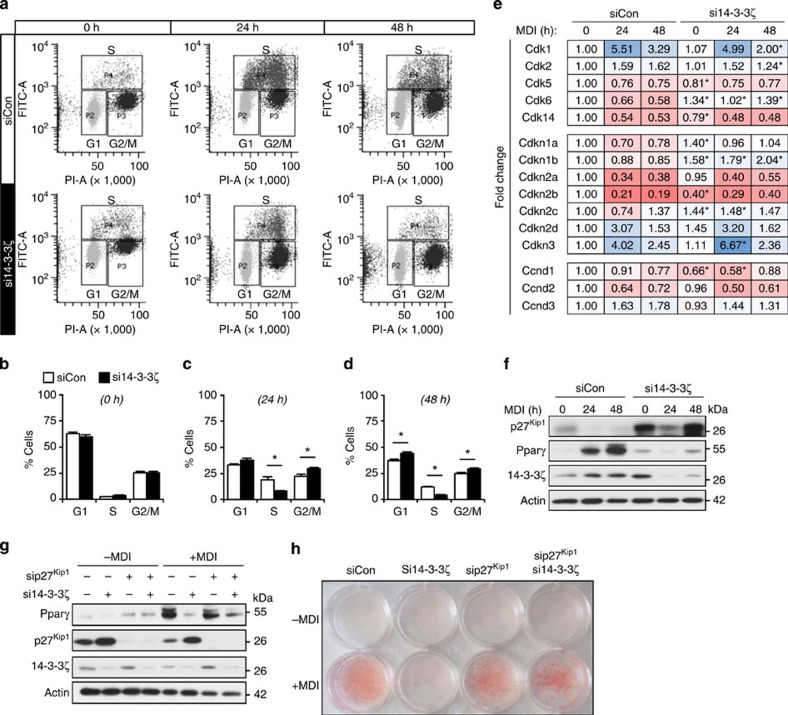
Defects in adipogenesis due to 14-3-3ζ depletion are due to cell cycle arrest and up-regulation of *Cdkn1b*/p27^Kip1^. (**a**–**d**) Analysis by flow cytometry (**a**) of control or si14-3-3ζ-transfected 3T3-L1 cells at different stages of the cell cycle at 0 (**b**), 24 (**c**) and 48 (**d**) hours following differentiation (*n*=4 independent experiments; *P<0.05 when compared with siCon-transfected cells, assessed by Student's *t*-test). (**e**) Analysis of various cell cycle regulatory genes by RNA-Seq from siCon- or si14-3-3ζ transfected 3T3-L1 adipocytes (*n*=4 per goup, *P<0.05 when compared with siCon cells at the same time point, assessed by Student's *t*-test). (**f**) Immunoblotting of p27^Kip1^ from lysates of differentiating of siCon- or si14-3-3ζ-3T3-L1 adipocytes (*n*=4 experiments). (**g**,**h**) Co-transfection of siRNA against 14-3-3ζ and p27^Kip1^ was used to examine whether knockdown of both proteins could restore differentiation, as determined by Pparγ expression (**g**) or Oil Red-O staining (**h**). (*n*=4 independent experiments). Error bars represent s.e.m.

**Figure 7:**
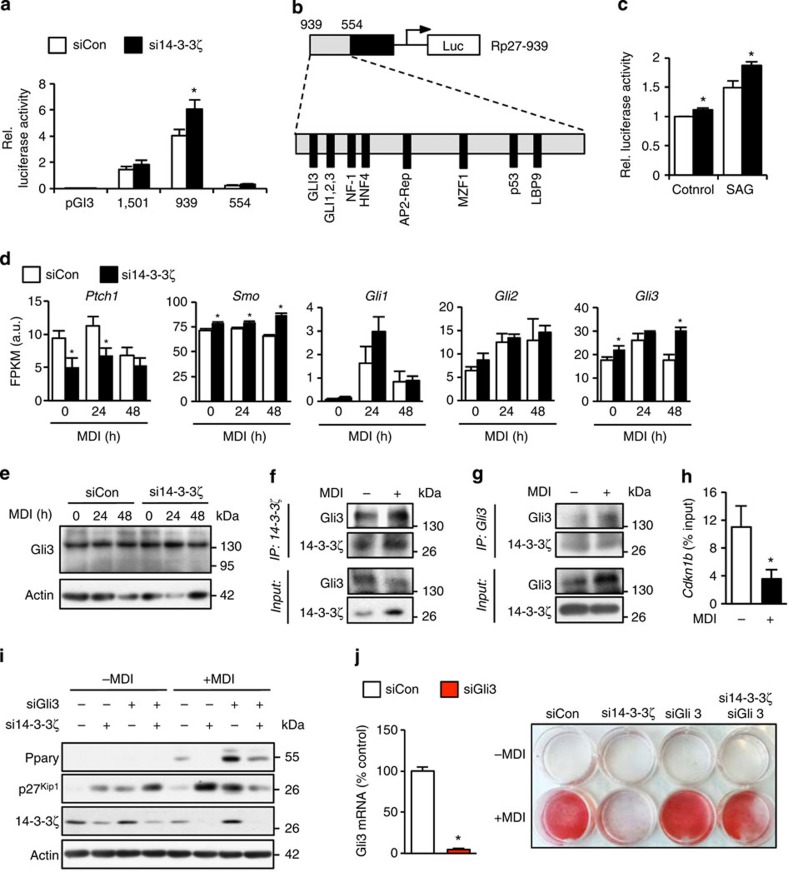
14-3-3ζ suppresses hedgehog signalling to control the expression of p27^Kip1^. (**a**) Measurement of relative luciferase activity from various lengths of the *Cdkn1b* promoter in control or 14-3-3ζ-depleted NIH-3T3 cells (*n*=7–8 per group, *P<0.05 when compared with control cells as assessed by Student's *t*-test). (**b**) Transcription factor binding sites within the −939–554 promoter region of *Cdkn1b*. (**c**) Hedgehog signalling (Gli-dependent) transcriptional activity in control or si14-3-3ζ-transfected Shh-light2 cells treated with 100 nM SAG. (*n*=4 per group, *P<0.05 when compared to control cells, as assessed by Student's *t*-test). (**d**) Expression levels of various components of the hedgehog signalling pathway in differentiating control and 14-3-3ζ-depleted 3T3-L1 cells, as measured by RNA-seq (*n*=4 per group, *P<0.05 when compared to control cells, as assessed by Student's *t*-test). (**e**) Immunoblotting of full length (∼170 kDa) and processed (∼80 kDa) Gli3 in 14-3-3ζ-depleted 3T3-L1 cells treated with MDI (representative of *n*=3 experiments). (**f**,**g**) Co-immunoprecipitation of endogenous (**f**) 14-3-3ζ or (**g**) Gli3 from lysates of 3T3-L1 cells treated with MDI for 24 h (representative of *n*=3 experiments) (**h**) ChIP-qPCR analysis of Gli3 occupancy on the *Cdkn1b* promoter in 3T3-L1 cells treated with MDI for 24 h (*n*=4 per group, *P<0.05, as assessed by Student's *t*-test). (**i**,**j**) Co-transfection of siRNA against 14-3-3ζ and Gli3 was used to examine whether knockdown of both proteins could restore differentiation, as determined by Pparγ expression (**i**) or Oil Red-O staining (**j**) (*n*=4 independent experiments). Error bars represent s.e.m.

To gain a broader understanding of how 14-3-3ζ depletion affects biological processes within the differentiating adipocyte, we first compared significantly changed genes at *t*=0, 24 and 48 h after differentiation (Fig. 5d, Supplementary Fig. 5a,b). Gene ontology classification of significantly differentially expressed genes revealed changes in various biological processes due to 14-3-3ζ knockdown. Gene-set enrichment analysis^32^ revealed that 14-3-3ζ knockdown significantly modulated multiple cell cycle genes (Supplementary Tables 3–8). We next investigated how 14-3-3ζ regulates the cell cycle, a key process in differentiating 3T3-L1 cells^33^, using flow cytometry. Knockdown of 14-3-3ζ led to an accumulation of cells at G1 phase during the first 48 h of differentiation (Fig. 6a–d). To further understand how depletion of 14-3-3ζ promoted cell cycle arrest, we examined the expression profiles of cell cycle regulatory genes. *Cdkn1b* and its product p27^Kip1^ were consistently upregulated in 14-3-3ζ-depleted cells (Fig. 6e,f). p27^Kip1^ controls the G1- to S-phase transition in murine pre-adipocytes^33^, and defects in adipogenesis were associated with increased p27^Kip1^ abundance during the critical period of differentiation (Fig. 6f). To determine whether the regulation of p27^Kip1^ by 14-3-3ζ during adipogenesis was required for adipogenesis, 3T3-L1 cells were co-transfected with siRNA against 14-3-3ζ and p27^Kip1^ (Fig. 6g,h; Supplementary Fig. 6a). Simultaneous knockdown of both proteins rescued the defect in adipocyte differentiation, as observed by Pparγ abundance and Oil Red-O incorporation (Fig. 7g,h; Supplementary Fig. 6b). The rescue of differentiation was specific to *Cdkn1b/*p27^Kip1^ knockdown, as depletion of *Cdkn1a* and *Cdkn2c* did not rescue the defect in adipogenesis (Supplementary Fig. 6d). These observations demonstrate that 14-3-3ζ functions upstream of the master adipogenic transcriptional programme and is required for the proper maintenance of cell cycle progression during adipogenesis.

### 14-3-3ζ inhibits hedgehog signaling to regulate Cdkn1b

Analysis of the *Cdkn1b* promoter revealed that 14-3-3ζ knockdown potentiated basal promoter activity of the region between 939 and 554 (Fig. 7a). Within this region, several binding motifs were identified for Gli proteins (Fig. 7b)^34^, which are hedgehog signalling effectors known to regulate adipogenesis^35^,^36^. Previous studies in other cell types have pointed to physical interactions between the 14-3-3ɛ isoform and Gli proteins^37^. Thus, we assessed whether there are physical and functional links between 14-3-3ζ and Gli proteins. Using Shh-light2 cells^38^ to measure Gli protein-dependent hedgehog activity, we found that 14-3-3ζ knockdown increased both basal and SAG-induced Gli protein-dependent activity (Fig. 7c). The observation that 14-3-3ζ knockdown increased *Cdkn1b* promoter activity and Gli-dependent hedgehog activity (Fig. 7a,c) prompted us to examine whether 14-3-3ζ regulated the expression of hedgehog effectors themselves. 14-3-3ζ knockdown decreased *Ptch1* expression and significantly increased *Smo* and *Gli3* expression (Fig. 7d). Gli3 can function as an activator or a repressor depending on proteolytic cleavage^39^, and knockdown of 14-3-3ζ did not promote the expression of the repressor form (Fig. 7e). Gli3 was found to complex with 14-3-3ζ during differentiation (Fig. 7f,g), and this was associated with decreased occupancy of Gli3 on the *Cdkn1b* promoter (Fig. 7h), suggesting that 14-3-3ζ restricts Gli3-dependent *Cdkn1b*/p27^Kip1^ expression. In undifferentiated cells, transfection with si14-3-3ζ or siGli3, or co-transfection of si14-3-3ζ and siGli3, upregulated p27^Kip1^ protein abundance. In cells treated with the differentiation cocktail for 48 h, transfection of siGli3 alone potentiated Pparγ protein abundance despite increased abundance of p27^Kip1^. Co-transfection of si14-3-3ζ and siGli3 reduced p27^Kip1^ protein abundance and permitted the induction of Pparγ and ultimately differentiation into a mature adipocyte (Fig. 7i,j). In contrast, knockdown of the closely related *Gli1* and *Gli2* genes did not rescue the defect in adipogenesis (Supplementary Fig. 7). Collectively, these rescue experiments clearly demonstrate that 14-3-3ζ regulates the expression of p27^Kip1^ and the adipocyte progenitor cell cycle through the hedgehog effector Gli3 to control adipocyte differentiation *in vitro*.

### 14-3-3ζ alters Gli3 and p27^Kip1^ expression in e18.5 embryos

**Figure 8:**
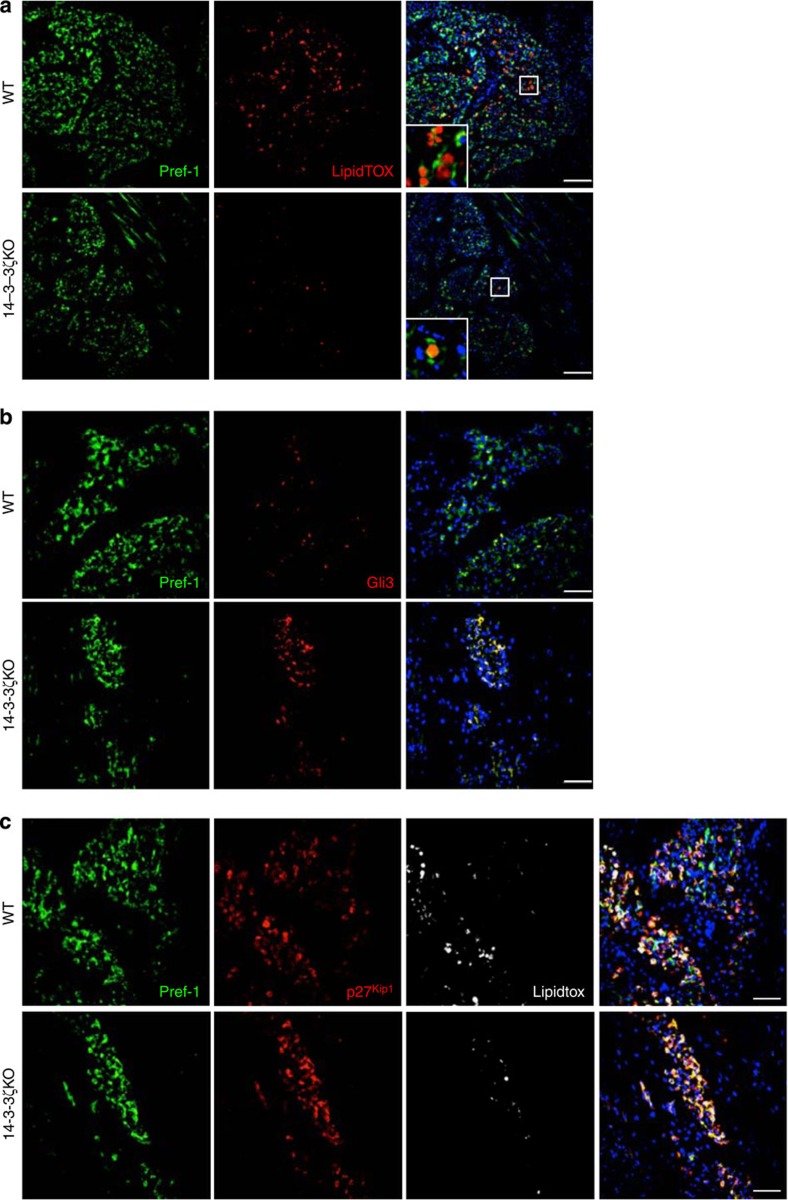
Deletion of 14-3-3ζ causes the aberrant expression of Gli3 and p27^Kip1^ in embryonic Pref-1-labelled adipose precursor cells. (**a**–**c**) Frozen sagittal sections of e18.5 embryos were stained for (**a**) Pref-1 alone to examine differences in the number of adipose precursor cells, (**b**) Pref-1 and Gli3, and (**c**) Pref-1 and p27^Kip1^. LipidTOX Deep-red was used to label mature adipocytes within the embryo (representative images of *n*=3 per genotype; Scale bar=10μm (**a**) or 50 μm (**b**,**c**)).

The number of adipocytes is established early in life, with minimal proliferation or apoptosis later in adults^40^. The mechanistic studies described above pointed to an early defect in adipogenesis, perhaps at the level of adipocyte precursors, in 14-3-3ζKO mice. Indeed, differences in adiposity could be due to the fact that 14-3-3ζKO mice are born either with a reduction in the number of adipose precursor cells or a specific group of precursors that cannot differentiate into mature adipocytes. Thus, we examined e18.5 stage embryos using Pref-1 as a marker for adipocyte precursors^41^. Qualitatively, the number of Pref-1 cells appeared to be slightly decreased (Fig. 8a). We observed striking reductions in the number of lipid-laden ‘mature' adipocytes in 14-3-3ζKO embryos (Fig. 8a). Adipose precursors in 14-3-3ζKO embryos displayed marked increases in Gli3 and p27^Kip1^ immunoreactivity (Fig. 8b,c), which complements our *in vitro* findings where depletion of 14-3-3ζ increased Gli3 and p27^Kip1^ expression in 3T3-L1 pre-adipocytes (Fig. 6f,g; Fig. 7d). Taken together, these findings suggest that *in vivo* deletion of 14-3-3ζ alters the expression of Gli3 and p27^Kip1^ in adipose precursors in developing embryos before birth and may help set life-long adiposity.

### Effects of high-fat feeding in 14-3-3ζKO mice

**Figure 9:**
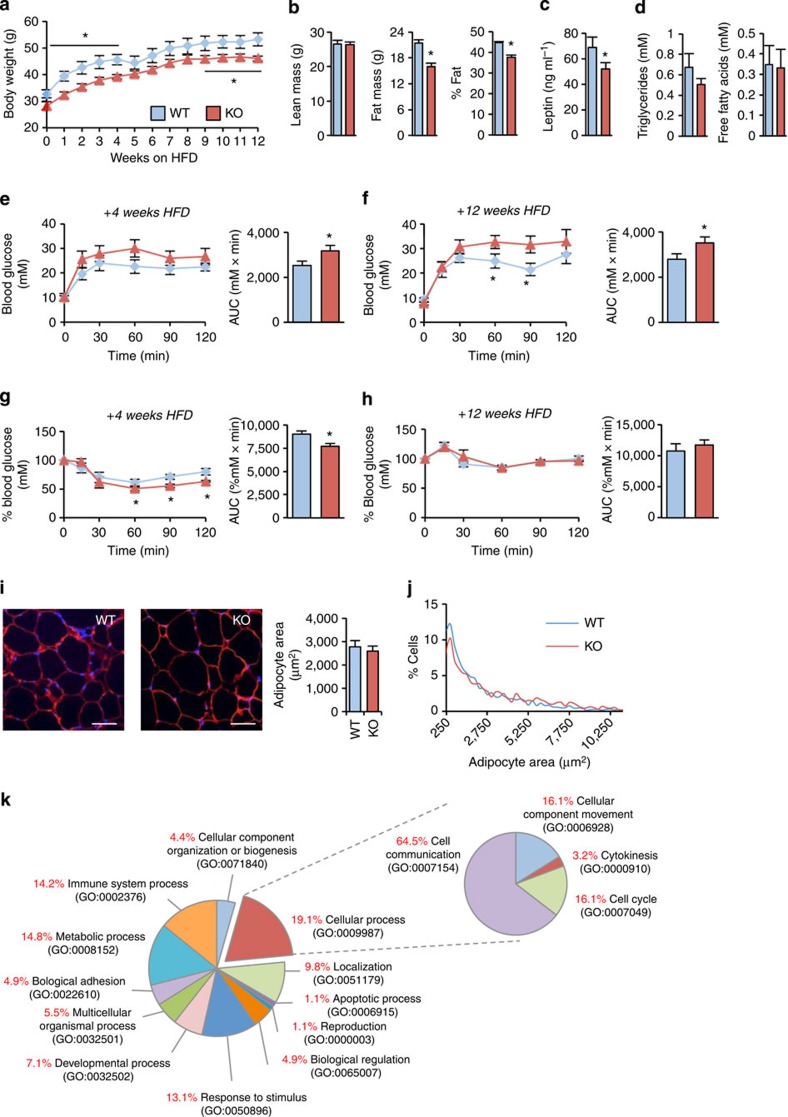
14-3-3ζKO mice can gain weight on a high-fat diet (**a**) Wild-type (WT) and 14-3-3ζKO mice were placed on a high-fat diet (60%) for 12 weeks (*n*=5 per group). (**b**) After 8 weeks, of high-fat feeding, WT and KO mice were subjected to DEXA body composition analysis (*n*=5 per group). (**c**,**d**) Plasma levels of (**c**) leptin (**d**) triglycerides and fatty acids were measured after high-fat feeding (*n*=5 per group). (**e**–**h**) WT and KO mice were administered glucose tolerance (**e**,**f**) and insulin tolerance (**g**,**h**) tests after 4 weeks (**e**,**g**) and 8 weeks (**f**,**h**) of high-fat diet exposure (*n*=5 per group). (**i**) Analysis of white adipocyte area from gonadal fat pads from 26 week old WT and KO mice (*n*=5 per group; Scale bar=100 μm). (**j**) Size distribution of adipocytes (*n*=5 per group). (**k**) Panther GO analysis of differentially expressed genes in gonadal white adipose tissue of WT and 14-3-3ζKO mice fed a low-fat diet (*n*=5–6 per genotype). Significant differences between WT and KO mice are indicated by *P<0.05 (assessed by Student's *t*-test). Error bars represent s.e.m.

Collectively, the data presented above establish that 14-3-3ζ plays a critical role in the differentiation of adipocyte progenitors towards a mature state. Next we assessed whether the adipocytes that do develop in 14-3-3ζKO mice were capable of expanding in response to a high-fat diet challenge. The differences in body weight, body composition and leptin were maintained between 14-3-3ζKO and wild-type mice during a 12-week 60% fat diet (Fig. 9a–c). No differences in fatty acid or triglyceride concentrations were observed (Fig. 9d). High-fat diet-fed 14-3-3ζKO mice were modestly glucose intolerant compared with wild-type controls (Fig. 9e–h). Analysis of white adipocyte morphology from 14-3-3ζKO gonadal fat pads revealed an expansion in size similar to that of wild-type mice fed the high-fat diet (Fig. 8i,j). Transcriptome analysis of adult gonadal white adipose tissue revealed only 78 genes that were significantly changed in 14-3-3ζKO mice (Supplementary Table 9), suggesting that fat from WT and 14-3-3ζKO under these conditions was relatively phenotypically normal. Both genotypes gained weight at a similar rate, suggesting that adaptive weight gain in 14-3-3ζKO mice is still possible via hypertrophy of existing adipocytes (Fig. 9i,j). As 14-3-3ζKO mice still maintain their differences in adiposity, it implies that the functional capacity of precursor cells to differentiate is reduced early in life.

## Discussion

**Figure 10:**
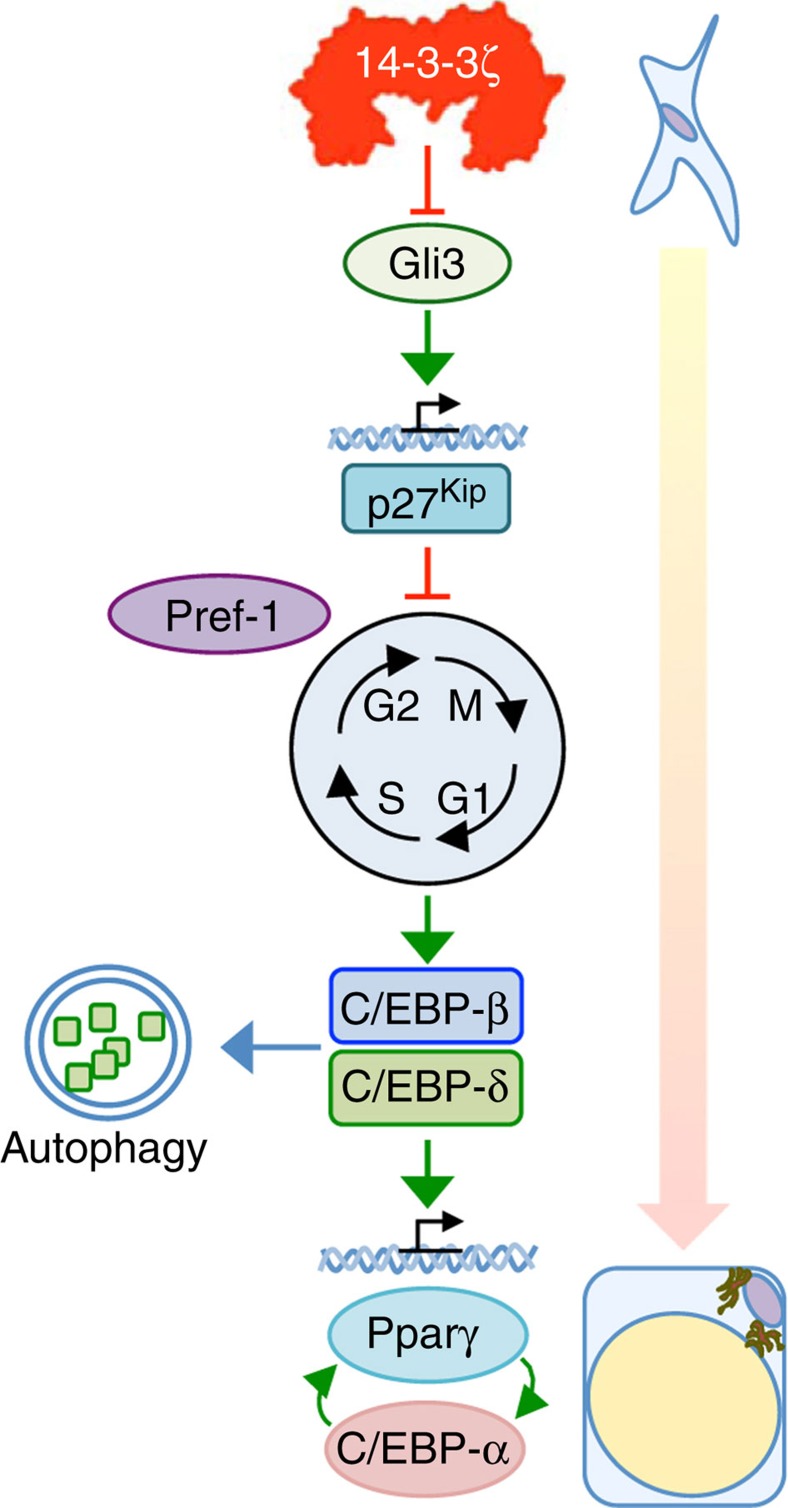
Working model of 14-3-3ζ-directed adipogenesis. During the early induction phase of adipocyte differentiation, 14-3-3ζ functions as a critical upstream regulator of adipogenesis as it controls mitotic clonal expansion through regulation of Gli3-dependent expression of the cyclin-dependent kinase inhibitor, *Cdkn1b*/p27^Kip1^. This permits the proper expression of Gli3 and p27^Kip1^ in adipose precursor cells in the developing embryo to generate function adipocyte precursors after birth. In the latter stages of the differentiation, 14-3-3ζ promotes the stability and translocation of C/EBP-δ into the nucleus where it is required for inducing the latent expression of the ‘master' transcription factors, Pparγ and C/EBP-α.

The goal of the present study was to understand the role of a 14-3-3 adaptor protein in obesity and energy homeostasis. We report that the 14-3-3ζ isoform is uniquely essential for full adipogenesis *in vitro* and *in vivo*. Mice lacking 14-3-3ζ had a visceral adipose depot-specific lean phenotype from birth and mild insulin resistance. Transgenic 14-3-3ζ overexpression led to the opposite phenotype, exhibiting age-related and high-fat diet-induced obesity without metabolic dysfunction. Mechanistic studies demonstrated that 14-3-3ζ regulates parallel proximal events underlying adipocyte differentiation, including the control of C/EBP-δ stability and cell cycle entry via hedgehog-dependent p27^Kip1^ expression (Fig. 10). Collectively, our findings reveal unexpected roles for 14-3-3ζ in pathways that govern adipocyte differentiation and demonstrate that elevated 14-3-3ζ expression alone is sufficient to drive obesity.

In the present study, we employed global knockout and overexpression mouse models, which provide information on the systemic effects 14-3-3ζ. However, without tissue-specific gene manipulation it is not possible to formally rule out potential contributions of non-adipocyte cell types and other tissues in the phenotype of these mice. Notwithstanding, our metabolic cage studies suggested that the decreased adiposity was not due to changes in food intake or whole-body energy expenditure, consistent with a primary role for direct effects on fat tissue. The robust defects in adipogenesis could be recapitulated in mouse embryonic fibroblasts derived from 14-3-3ζKO mice and 3T3-L1 and 3T3-F442A pre-adipocytes, which points to cell autonomous effects of 14-3-3ζ in these *in vitro* adipocyte models. Comparing the differentiation of control and 14-3-3ζKO adipose precursors cells transplanted into wild-type mice^42^ would conceivably translate our *in vitro* observations into an *in vivo* context, but we estimate that such an experiment would require up to 25 14-3-3ζKO donor mice, which represents a nearly insurmountable challenge given the low breeding efficiency in our colony. Moreover, such an experiment would not rule out subtle effects of other organ systems on the overall phenotype of the 14-3-3ζKO mice. Collectively, our data point to adipocyte-centred effects of 14-3-3ζ, but it will be important to assess this directly in future studies once adipocyte-specific 14-3-3ζKO mice become available.

Another limitation of our study is that we were unable to determine the precise stage(s) when 14-3-3ζ acts on adipogenesis and glucose homeostasis *in vivo* without temporal control of our gene manipulations. 14-3-3ζKO mice were runted at birth and exhibited rapid catch-up growth. Given that catch-up growth is associated with metabolic disease^43^,^44^, some of the minor effects on glucose homeostasis and insulin sensitivity in 14-3-3ζKO mice may result from aberrant fetal programming. However, the primary phenotype stemming from systemic deletion of 14-3-3ζ was decreased adiposity, rather than robust changes in glucose homeostasis.

The decreased adiposity from birth observed with *in vivo* 14-3-3ζ deletion suggests that 14-3-3ζ may predetermine the number of maturing adipocyte precursors during development to influence adiposity in adulthood^40^. Subcutaneous adipose tissue is thought to develop embryonically, whereas gonadal adipose tissue is thought to develop postnatally^45^,^46^, and while we observed striking reductions in the number of mature adipocytes in 14-3-3ζKO embryos, it is unclear which depots they will correspond to postnatally. 14-3-3ζKO mice still gained weight when challenged with a high-fat diet, suggesting that the developmental effects of 14-3-3ζ can be uncoupled from pathways controlling adipocyte size in adults. Thus, adipocyte precursors in adult 14-3-3ζKO mice, if they play a role in increased adipocyte tissue size, are likely to have intrinsic gene networks that are independent of the functions of 14-3-3ζ (refs 3, 46, 47). It is a limitation of our study that we did not quantify the absolute number of adipocytes in fat pads in our mice, and therefore the relative roles of hyperplasia versus hypertrophy are uncertain in our models.

Molecular adaptors have not been well studied in the context of obesity or glucose homeostasis. Knockdown of 14-3-3β has previously been reported to impair 3T3-L1 differentiation due to defects in lipid storage processes^48^, but we were unable to replicate its requirement using our siRNAs validated not to affect other 14-3-3 isoforms. In contrast, we found a unique role for 14-3-3ζ in controlling the induction of the master adipogenic transcription factors Pparγ and C/EBP-α, which are required for the expression of genes involved in lipid uptake and storage. Pparγ and C/EBP-α are both dependent on the actions of and activation of C/EBP-β and C/EBP-δ (refs 6, 7). Depletion of 14-3-3ζ promoted autophagy-dependent degradation of C/EBP-δ, but additional studies are warranted to understand how 14-3-3ζ controls C/EBP-δ stability, as neither protein was found to direct interaction with the other. We did observe interactions of 14-3-3ζ with C/EBP-β during differentiation, which suggests a novel mechanism by which 14-3-3ζ may exert its effects on adipogenesis. Before binding to the Pparγ promoter, C/EBP-β is known to form macromolecular complexes consisting of transcription factors, coregulators and 14-3-3θ (refs 49, 50). As 14-3-3 proteins form heterodimers^9^,^10^, 14-3-3ζ may dimerize with 14-3-3θ to aid in the formation of these complexes to drive the expression of Pparγ. It should be clearly noted that it remains unclear whether the interaction between C/EBP-β and 14-3-3ζ is direct or indirect, and it is not known whether C/EBP-β harbours the canonical phosphorylation motifs that promote binding^9^,^10^. We also found that 14-3-3ζ controls the expression of other genes reported to be required for adipogenesis, such as *G0s2* and *Arxes*^30^,^31^. Our data point to multiple roles for 14-3-3ζ in the highly regulated process of adipogenesis.

We also identified a novel role for 14-3-3ζ in adipocyte progenitor cell cycle progression. Adult human pre-adipocytes, derived from subcutaneous adipose tissue, are not thought to undergo mitotic clonal expansion before undergoing adipogenesis^51^,^52^, but it remains unclear whether this also applies to fetal pre-adipocytes and/or pre-adipocytes from other depots. Murine pre-adipocytes enter the cell cycle during differentiation via the rapid turnover of p27^Kip1^ to promote the expression of C/EBP-β and C/EBP-δ^7^,^33^. Depletion of 14-3-3ζ *in vitro* and *in vivo* promoted the aberrant expression of *Cdkn1b/* p27^Kip1^, which prevented adipogenesis *in vitro*. Examination of the *Cdkn1b* promoter revealed binding sites for Gli transcription factors, which are established effectors of the hedgehog signalling pathway and known to interact with 14-3-3 proteins^36^,^37^. Activation of hedgehog signalling attenuates adipogenesis^35^,^53^, but the downstream effectors that mediate these effects have yet to be fully elucidated. Gli proteins are required for development and organogenesis^39^,^54^, and their ability to function as transcriptional activators or repressors^39^,^54^ made them likely candidates to mediate the inhibitory actions of hedgehog signalling on adipogenesis. Our findings directly implicate Gli3 in this process *in vitro*, as knockdown of 14-3-3ζ potentiated Gli protein-dependent transcriptional activity and Gli3 depletion was sufficient to restore adipogenesis in 14-3-3ζ-depleted cells. This places 14-3-3ζ upstream of hedgehog signalling, p27^Kip1^-dependent cell cycle progression and ultimately adipogenesis. The *in vivo* function of Gli3 in the regulation of adipogenesis requires further study, but we observed co-localization of Gli3, in addition to p27^Kip1^, in all Pref-1-marked adipocyte precursor cells in 14-3-3ζKO embryos^41^. Taken with our *in vitro* findings that demonstrate inhibitory actions of Gli3 and p27^Kip1^, this suggests that adipose precursors present in adult 14-3-3ζKO mice may have acquired their defect in differentiation during embryogenesis. Furthermore, this raises the possibility of similar defects in human pre-adipocytes during embryo development. Rescue studies employing new transgenic and compound knockout animals will be required to confirm that the Gli3/p27^Kip1^ axis is mechanistically downstream of 14-3-3ζ *in vivo*.

Pharmacological interventions for obesity have been developed, but their effectiveness and efficacy have been limited^55^,^56^. In obese individuals, expression of 14-3-3ζ and other isoforms has been shown to be elevated in visceral and subcutaneous adipose tissue depots^17^,^18^,^19^, but whether these changes in 14-3-3 protein expression are causal or associative was not known. Our data suggest that 14-3-3ζ overexpression exacerbates age-related and diet-induced obesity, independent of changes in glucose tolerance, insulin sensitivity or lipid profile. This suggests that 14-3-3ζ is a novel factor that may preferentially drive the expansion of metabolically healthy adipocytes^4^,^5^. It should be noted that 14-3-3ζKO and 14-3-3ζ-overexpressing mice have different genetic backgrounds, precluding direct comparisons between strains. Background strain differences may affect differences in glucose tolerance, insulin sensitivity or weight gain, but do not invalidate the within-model comparisons that involved strict littermate controls. Further studies are required to examine the potential obesogenic effect 14-3-3ζ overexpression on other genetic backgrounds^57^,^58^.

In conclusion, results from this study demonstrate, for the first time, essential roles for 14-3-3ζ in adipogenesis. Our data add additional levels of complexity to our current understanding of adipocyte differentiation, as one must now consider the function of 14-3-3 proteins and other types of molecular adaptors during adipogenesis. Aside from its ability to enhance the expression of the key adipogenic transcription factors, our transcriptomic analysis revealed the requirement for 14-3-3ζ in regulating the expression of key genes involved in adipocyte differentiation. This indicates that 14-3-3ζ has critical roles in the development of mature visceral white adipocytes and that adaptor proteins from the 14-3-3 family can therefore act as specific master regulators of cell differentiation by controlling diverse processes. Our study demonstrates the presence of a 14-3-3ζ-Gli3-p27^Kip1^ axis that regulates adipocyte differentiation and suggest that targeting components of this axis may be a beneficial therapeutic approach for the treatment of obesity.

## Methods

### Animal husbandry and metabolic analyses

Male 14-3-3ζ knockout mice on a C57/BL6 background^9^ and 14-3-3ζTAP transgenic mice on a CD1 background^22^ were housed in a specific pathogen-free facility at the University of British Columbia in a 12:12 light: dark, temperature and humidity controlled environment. On a pure C57/BL6 background, heterozygous breeding of mice with 14-3-3ζ null alleles did not yield progeny at the expected Mendelian ratio. Littermate controls were used in all experiments. For glucose- and insulin-tolerance tests, 14-3-3ζKO or 14-3-3ζTAP male mice at 10 and 25 weeks or 9 and 52 weeks, respectively, were fasted for 6 h, followed by i.p. injection of 2 g kg^−1^ glucose or 1.5 U kg^−1^ Humalog insulin (Eli Lilly, Indianapolis, IN), respectively. Tail vein blood glucose levels were measured with a glucometer (OneTouch UltraMini, Life Scan, Milpitas, CA). Commercially available kits were used to measure plasma levels of insulin, adiponectin and corticosterone (Alpco, Salem, NH); leptin (Crystal Chem, Downer's Grove, IL); and triglycerides and free fatty acids (Biovision; Milpitas, CA). Body composition was measured by dual-energy x-ray absorptiometry (DEXA) with a PIXImus Mouse Densitometer (Inside Outside Sales, Madison, WI). Mice were also fed *ad libitum* a 60% fat diet or its respective 10% control diet (Research Diets, New Brunswick, NJ) for 8 or 12 weeks. Food intake and energy expenditure were measured for 72 h using PhenoMaster metabolic cages (TSE Systems, Bad Homburg, Germany), after 1-week acclimation. Data were averaged from the last two full light:dark cycles. All procedures were approved by the University of British Columbia Committee on Animal Care in accordance with international guidelines.

### Cell culture and transient transfections

3T3-L1 pre-adipocytes (ZenBio; Research Triangle Park, NC), 3T3-F442A cells^25^ (provided by Dr A. Sorisky, Ottawa Health Research Institute, Ottawa, Canada), NIH-3T3 cells, mouse embryonic fibroblasts (MEFs) from WT and 14-3-3ζKO mice, and Shh-Light2 cells^38^ (provided by Dr V. Wallace, University of Toronto, Toronto, Canada) were maintained in 25 mM glucose DMEM, supplemented with 10% newborn calf serum and 1% penicillin/streptomycin. 3T3-L1 cells were differentiated by allowing cells to reach confluence, followed by a cocktail (MDI) of 172 nM insulin, 500 μM IBMX and 500 nM dexamethasone in differentiation media containing 25 mM glucose DMEM and 10% fetal bovine serum. MEFs were differentiated with differentiation media supplemented with 5 μM rosiglitazone (Sigma-Aldrich, St Louis, MO). 3T3-F442A cells were differentiated in differentiation media supplemented with 5 μg ml^−1^ insulin. Two days following induction, cells were maintained in differentiation media with insulin, and after 7 or 14 days, cells collected for RNA or protein or stained with Oil Red-O to assess adipogenesis. Cycloheximide, MG132 or epoxomicin, or 3-methyladenine or chloroquine (Sigma-Aldrich) were used to inhibit protein translation, the proteasome or autophagy, respectively. To inhibit all 14-3-3 proteins, 3T3-L1 pre-adipocytes were pretreated with 14-3-3 Antagonist I,2-5 (14-3-3i, EMD Millipore, Bilerica, MA) that disrupts the interaction of 14-3-3 isoforms with their ligands^24^. Lipofectamine RNAiMax or 3000 was used to transfect cells with validated silencer select pre-designed siRNA (Life Technologies, Burlington, ON, Canada) or plasmids, respectively.

### Measurement of *Cdkn1b* and Gli-dependent promoter activity

NIH-3T3 cells were co-transfected with plasmids containing *Cdkn1b* promoter constructs of various lengths upstream of firefly luciferase (provided by Dr D. Everly, Rosalind Franklin University of Medicine and Science, North Chicago, IL)^59^ and *Renilla* luciferase (10:1 dilution), followed by transfection of siRNA against 14-3-3ζ or the scrambled control. To determine hedgehog-dependent transcriptional activity, Shh-light2 cells were treated with the synthetic Smoothened agonist (SAG; Cayman Chemicals, Ann Arbor, MI) or transfected with siRNA against 14-3-3ζ or the scrambled control. Luciferase activity was measured after 24 or 48 h with the Dual-Luciferase Reporter Assay system (Promega, Madison, WI). Transcription factor binding sites were analysed with MotifMap^34^. ChIP analysis of Gli3 binding to the *Cdkn1b* promoter was performed with antibodies against Gli3 and the Pierce Magnetic ChIP kit, as per the manufacturer's protocol (Thermo Scientific, Rockford, IL).

### Fluorescent microscopy and flow cytometry

Adipose tissue was harvested and fixed in 4% paraformaldehyde. Adipocyte size was measured by staining for perilipin in 5-μm-thick sections (Cell Signalling Technology, Danver, MA). Embryos at e18.5 were harvested from timed-pregnant dams, sexed by tail clip PCR^60^, fixed for 24 h in 4% paraformaldehyde, followed by storage in 70% ethanol, flash-frozen and sectioned to 8-μm-thick sections. Embryo sections were stained with antibodies (1:100 dilution) raised against Pref-1/DLK1 (C-19, sc-8624), Gli3 (H-280, sc-20688) or p27^Kip1^ (C-10, sc-528) (Santa Cruz). All sections were stained with appropriate host-derived Alexafluor-conjugated secondary antibodies (Life Technologies, diluted 1:2000), and when necessary, stained with LipidTOX Deep-red (diluted 1:200, Life Technologies) for the visualization of mature adipocytes. DAPI was used to visualize nuclei. Images of identical exposure times were taken with a Zeiss 200M inverted microscope. Cell size was measured with CellProfiler^61^.

Flow cytometry was performed on 3T3-L1 cells transfected with a scrambled control or siRNA against 14-3-3ζ, then induced to differentiate with MDI and harvested at 0, 24 and 48 h for, as previously described^62^, on a LSR II-561 Flow Cytometer (BD Biosciences, San Jose, CA). Quantification of cells at various stages of the cell cycle was performed by FlowJo software (v.10, Treestar, Ashland, OR).

### Immunoblotting and protein detection

Cells or tissues were lysed in RIPA buffer, supplemented with protease and phosphatase inhibitors, and in some instances prepared for cytosolic and nuclear fractionation, in accordance with manufacturer's protocols (Thermo Scientific). Immunoprecipitation was performed on whole-cell lysates from 3T3-L1 adipocytes at different stages of differentiation with established protocols^14^. Proteins were resolved by SDS–PAGE for detection, and PVDF membranes were probed with antibodies against 14-3-3ζ (#7413), C/EBP-α (D56F10, #8178), C/EBP-β (#3082), C/EBP-δ (#2318), Foxo1 (L27, #9454), Lipin1 (#5195), Pparγ (81B8, #2443), cleaved caspase-3 (5A1E, #9664), Pgc1-α (#4259), tubulin (#2146) and Lamin A/C (4C11, #4777) (all antibodies diluted 1:1,000, Cell Signaling Technology); p27^Kip1^ and Gli3 (all antibodies diluted 1:200; Santa Cruz Biotechnology, Santa Cruz, CA); TAP (CAB1001) and Gli3 (PA5-28029) (all antibodies diluted 1:1,000, Thermo Scientific) and β-actin (AC-15, #NB600-501) (Novus Biologicals, Littleton, CO). Original scans of immunoblots are shown in Supplementary Fig. 8.

### RNA isolation, quantitative real-time PCR and transcriptome analysis

RNA was isolated from mouse tissues or 3T3-L1 adipocytes with the RNEasy kit (Qiagen, Mississauga, ON, Canada). Transcript levels of synthesized cDNA (Quanta Biosciences, Gaithersburg, MD) were measured with SYBR green chemistry on a StepOnePlus Real-time PCR System (Life Technologies). All data were normalized to HPRT by the 2^−ΔCt^ method as described by Livak and Schmittgen^63^. Libraries for RNA-Seq were generated from isolated RNA, as recommended by the manufacturer's protocol (Illumina, Carlsbad, CA). Following pooling of sequence-indexed libraries, sequencing was performed on a HiSeq 2500 (Illumina) collecting 20 million paired-end reads (150 bp x2). Alignment of reads to the mouse genome (Ensembl NCBIM37) and analysis of differentially expressed genes (0.05 FDR-adjusted q<0.05) were performed by TopHat software (v.2.0.11) and the Cufflinks package (v.2.2.0), respectively^64^. Panther and gene-set enrichment analysis (GSEA) were performed on all RNA-Seq results used to examine gene sets or biological processes that were significantly enriched^32^,^65^.

### Statistical analysis

All data are expressed as the mean±s.e.m. Data were analysed by ANOVA followed by Dunnett or Bonferroni *t*-tests, or by Student's *t*-tests, and significance was achieved when *P*<0.05. A minimum of *n*=3 independent experiments was performed for analysis.

## Additional information

**How to cite this article:** Lim, G. E. *et al*. 14-3-3ζ coordinates adipogenesis of visceral fat. *Nat. Commun.* 6:7671 doi: 10.1038/ncomms8671 (2015).

## Supplementary Material

Supplementary Information Supplementary Figures 1-8 and Supplementary Tables 1-9
