# Supplementary material for: 14-3-3ζ coordinates adipogenesis of visceral fat
Source: Nat Commun. 2015 Jul 29;6:7671. doi: 10.1038/ncomms8671 (PMC4532800; doi:10.1038/ncomms8671)
Supplement: Supplementary Information — Supplementary Figures 1-8 and Supplementary Tables 1-9 [file ncomms8671-s1.pdf]

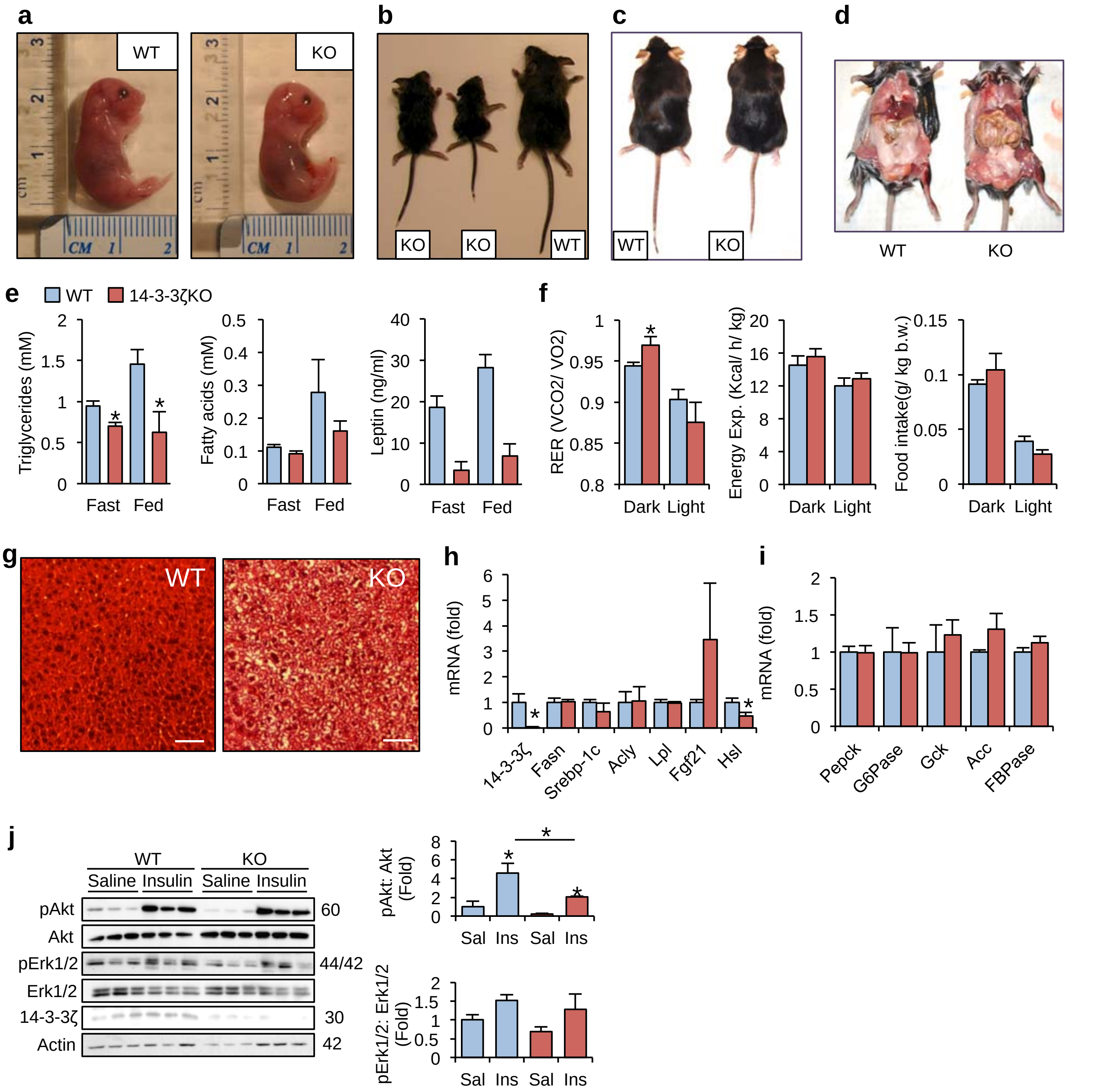

Supplementary Figure 1. 14-3-3ζKO mice display mild perturbations in glucose homeostasis and insulin sensitivity. (a-c) Images of 14-3-3ζKO embryos at e19.5 (a), mice at 3 weeks of age (b), and 24 weeks of age (c). **(d)** View of gonadal fat pads from WT and 14-3-3ζKO mice (representative of n=4 per genotype). **(e)** Fasting and random-fed plasma leptin, free fatty acid, and triglyceride levels from 32 week-old mice (n=5-7 per group). **(f)** Wild-type (WT) and 14-3-3ζKO mice were allowed to acclimate for 1 week prior to placement in metabolic cages to measure energy expenditure and food intake. Data were averaged from the last two full light: dark cycles (n=5-7). **(g)** Histological examination of livers showed marked steatosis in 14-3-3ζKO mice (representative of n = 6 mice per genotype). **(h,i)** Isolated RNA was subjected to quantitative PCR analysis of lipogenic (h) and gluconeogenic (i) genes between wildtype and 14-3-3ζKO mice (n=6 per genotype). **(j)** Akt phosphorylation, as measured by immunoblotting, 5 minutes after an *i.p.* bolus of insulin (5 U/kg) in liver samples from overnight-fasted WT and 14-3-3ζKO mice (n=6 per genotype). Significant differences, as assessed by Student's t-test or ANOVA, are indicated by *: $p<0.05$. Error bars represent S.E.M.

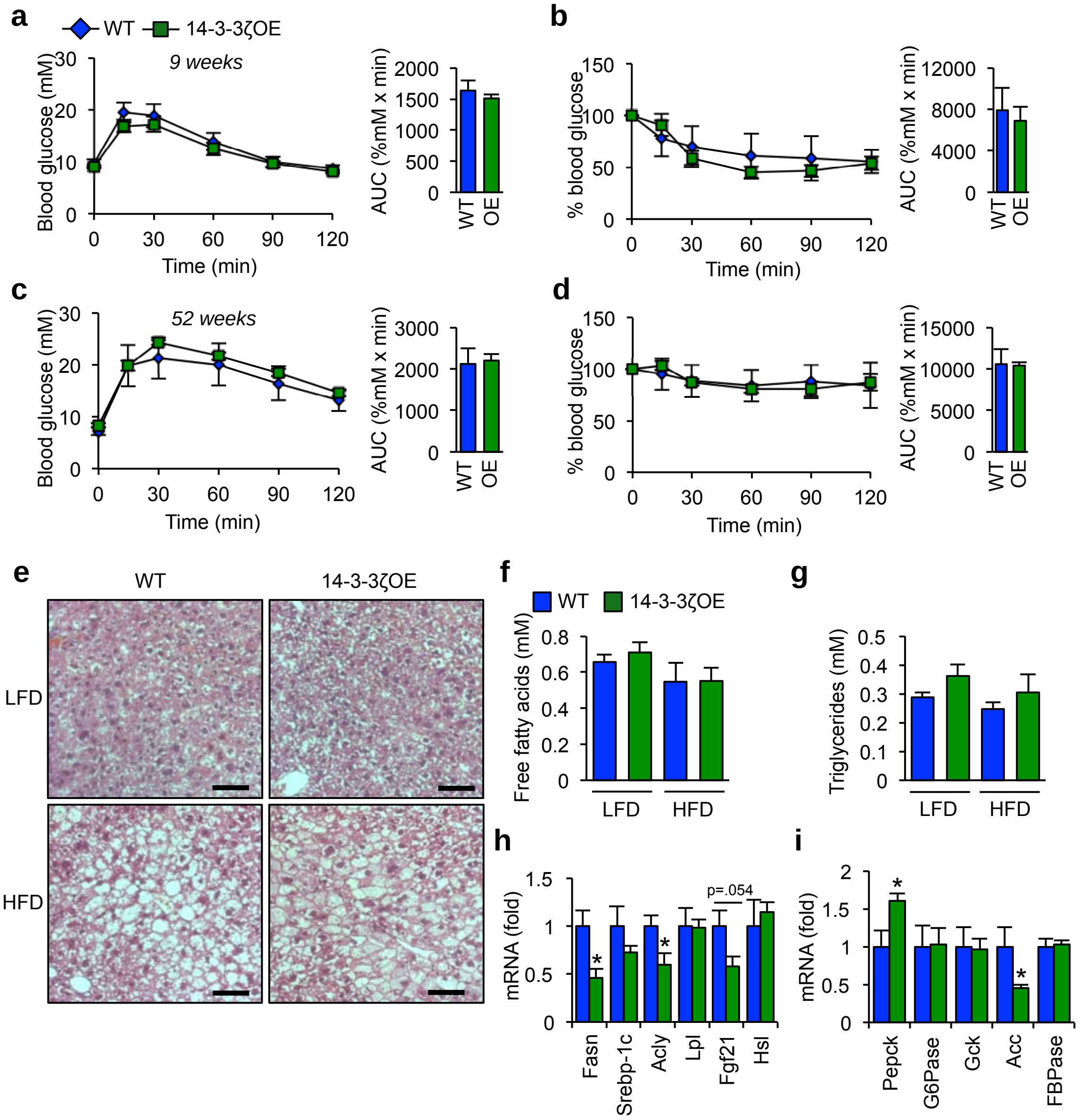

Supplementary Figure 2. Modest over-expression of 14-3-3ζ has minor effects on the liver. (a-d) Glucose tolerance (a,c) and insulin tolerance (b,d) tests were administered to WT and 14-3-3ζOE mice at 9 (a,b) and 52 (c,d) weeks of age (n= 4-9 per group). **(e)** Liver histology of wild-type (WT) and 14-3-3ζ over-expressing (14-3-3ζOE) mice fed low-fat (LFD, 10%) or high-fat (HFD, 60%) diets for 8 weeks (representative images of n=4 mice per group, scale bars= 100 μm). **(f,g)** Circulating fasting plasma free fatty acids (f) and triglycerides (g) from WT and 14-3-3ζOE mice (n=4-9 per group). **(h,i)** Isolated liver RNA was subjected to quantitative PCR analysis for lipogenic (h) and gluconeogenic (i) genes between WT and 14-3-3ζOE mice (n=4-9 per group). Significant differences, as assessed by Student's t-test, are indicated by *: $p<0.05$. Error bars represent S.E.M.

a

MDI (hr)	mRNA (Fold change) 14-3-3σ	14-3-3η	14-3-3γ	14-3-3β	14-3-3ε	14-3-3θ	14-3-3ζ
0	1.0 ± 0.1	1.0 ± 0.1	1.0 ± 0.1	1.0 ± 0.1	1.0 ± 0.1	1.0 ± 0.1	1.0 ± 0.1
24	0.8 ± 0.2	1.2 ± 0.1	1.4 ± 0.2	1.2 ± 0.2	1.8 ± 0.2*	1.8 ± 0.2*	1.6 ± 0.2*
48	1.1 ± 0.3	0.9 ± 0.4	1.5 ± 0.4	1.3 ± 0.1	1.0 ± 0.1	1.1 ± 0.1	1.6 ± 0.1*

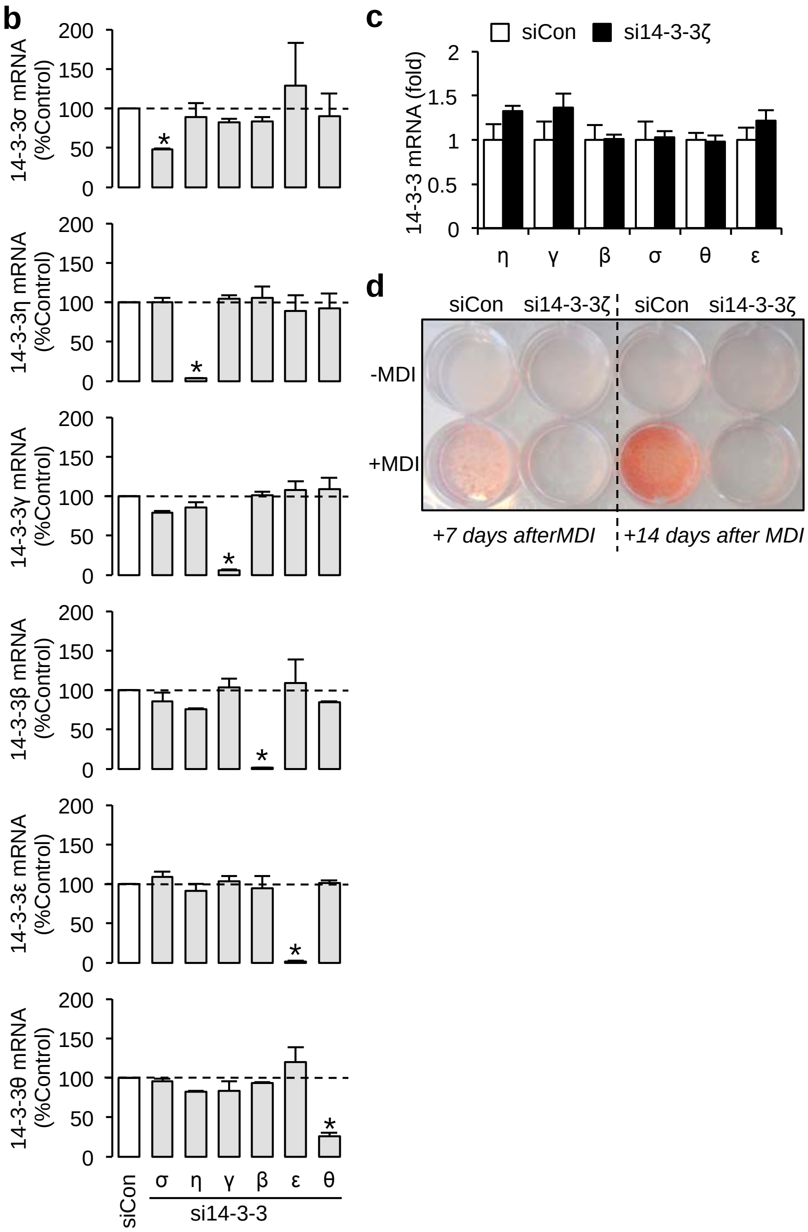

Supplementary Figure 3. Knockdown of 14-3-3ζ does not affect the expression of other isoforms in 3T3-L1 pre-adipocytes. (a) Expression profile of all 14-3-3 isoforms in 3T3-L1 pre-adipocytes during the first 48 hours of differentiation (n=4 independent experiments; *: p<0.05 when compared to undifferentiated cells, as assessed by one-way ANOVA). **(b)** 3T3-L1 pre-adipocytes were transfected with siRNA to knock down each mammalian 14-3-3 isoform. 48 hours after transfection, RNA was isolated, cDNA synthesized, and quantitative PCR was performed. All data were normalized to HPRT and quantified by the $2^{-\Delta C(t)}$ method (n=3 biological replicates, *: p<0.05 when compared to siCon, as assessed by Student's t-test). **(c)** Quantitative PCR analysis of remaining 14-3-3 isoforms from 14-3-3ζ-depleted 3T3-L1 pre-adipocytes (n=3 independent experiments). **(d)** Following transfection with siRNA and induction of differentiation with MDI, 3T3-L1 cells were maintained in insulin-containing media for 7 or 14 days (representative of n=3 experiments). Error bars represent S.E.M.

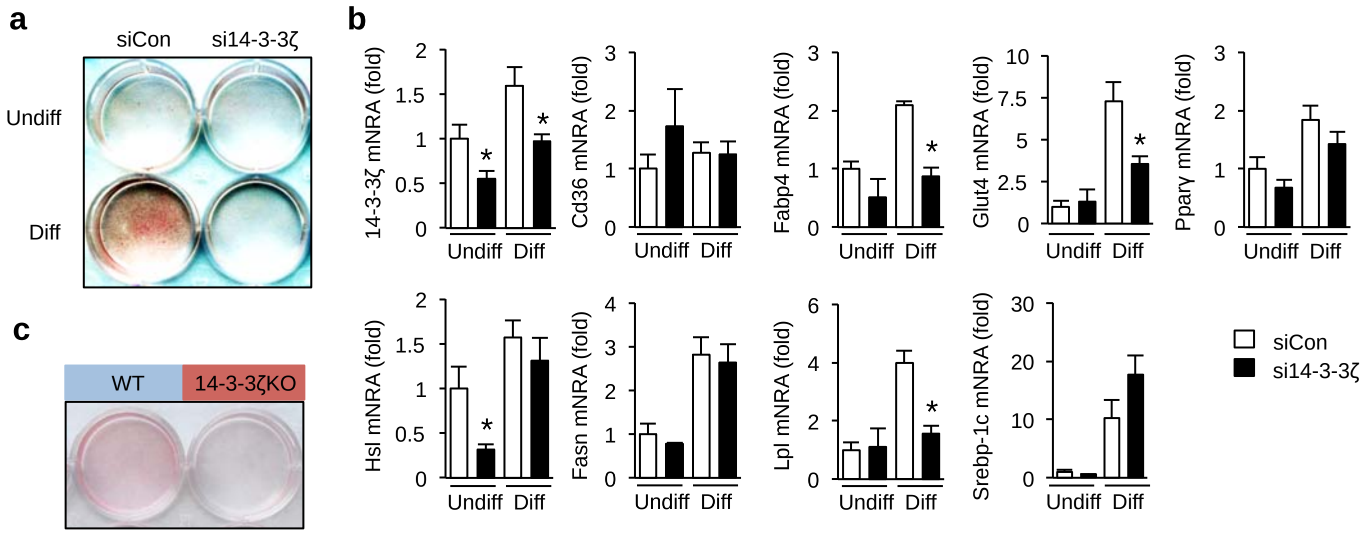

Supplementary Figure 4. Role of 14-3-3ζ in adipogenesis can be reproduced in 3T3-F442A cells and mouse embryonic fibroblasts. **(a)** 3T3-F442A cells were transfected with siRNA against 14-3-3ζ or the scrambled control (siCon) 24 hours prior to differentiation. Seven days after differentiation, cells were stained with Oil Red O (representative of n=3 independent experiments). **(b)** Quantitative PCR for mature adipocyte markers was performed on isolated RNA from transfected 3T3-F442A cells seven days following differentiation (n=4 biological replicates). **(c)** Differentiation of mouse embryonic fibroblasts derived from WT and 14-3-3ζKO mice into adipocytes (representative of n=4 independent experiments). Significant differences between siCon vs. si14-3-3ζ, as assessed by Student's t-test, are indicated by *: $p<0.05$. Error bars represent S.E.M.

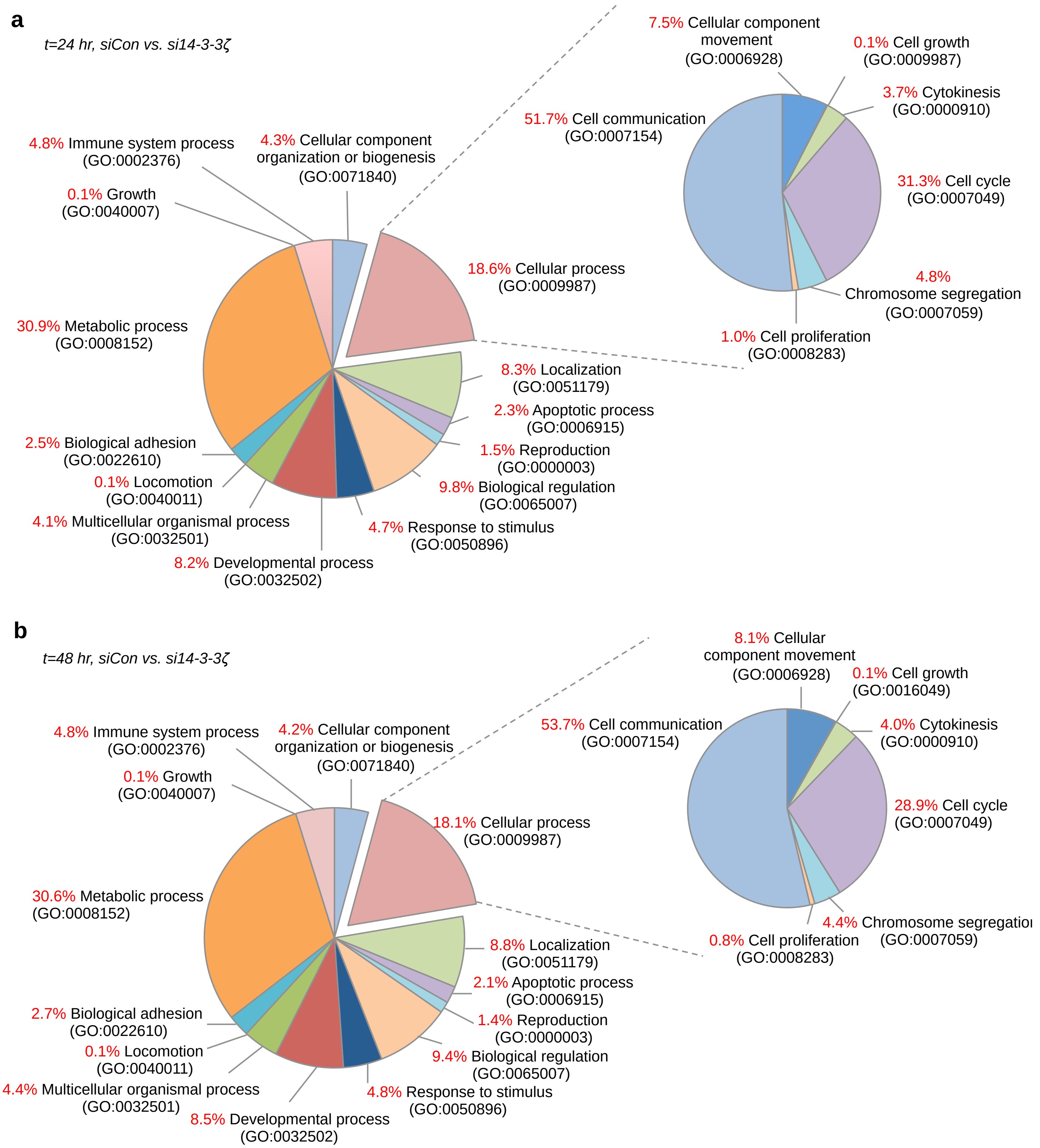

Supplementary Figure 5. Knockdown of 14-3-3ζ alters the transcriptome of differentiating 3T3-L1 adipocytes at 24 and 48 hours. (a-b) Panther gene ontology analysis was used to categorize transcriptome results of differentially expressed genes from 3T3-L1 adipocytes after 24 (a) and 48 (b) hours after differentiation. (RNA-Seq data were derived from n=5-6 mice per genotype).

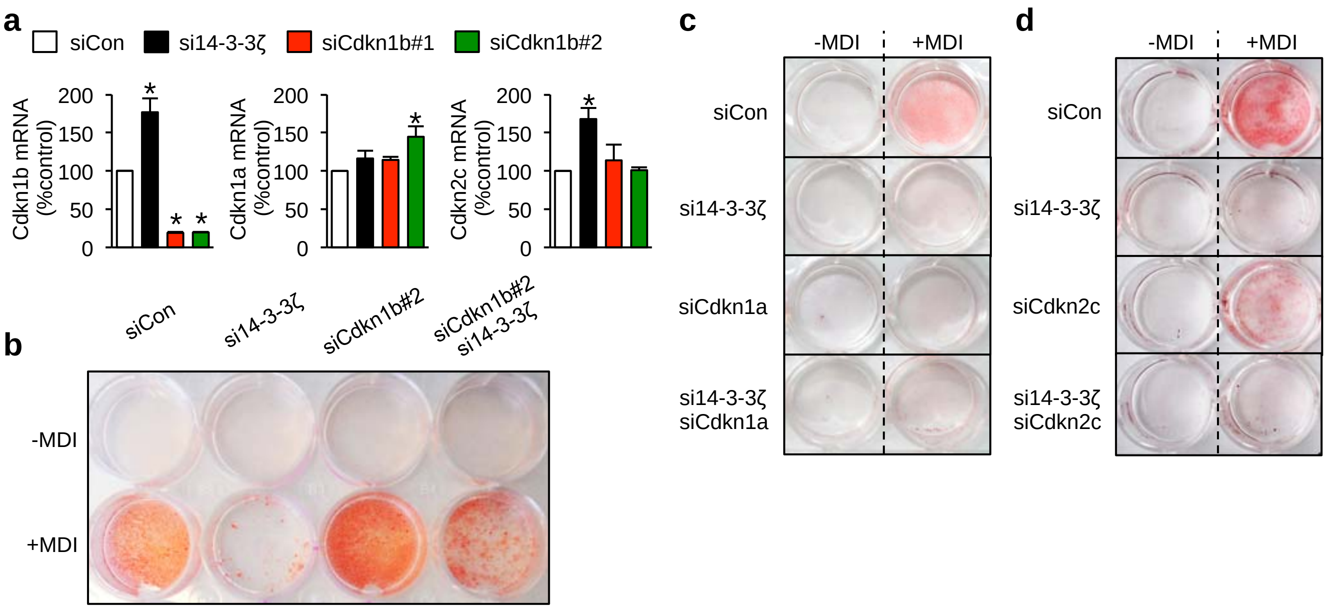

Supplementary Figure 6. Knockdown of only *Cdkn1b*/p27^{Kip1} was able to rescue the defect in differentiation of 14-3-3ζ-depleted cells. **(a)** Two distinct siRNA duplexes were used to knockdown *Cdkn1b* in 3T3-L1 pre-adipocytes. Due to off-target effects on siCdkn1b#2 on *Cdkn1a* expression, only siCdkn1b#1 was used (n=4 per group, *: p<0.05 when compared to siCon). **(b)** Co-transfection of siRNA against 14-3-3ζ and p27^{Kip1} (siCdkn1b#2) was used to examine if knockdown of both proteins could restore differentiation, as determined by Oil Red-O staining (h). (n=4 independent experiments). **(c,d)** Co-transfection of siRNA against 14-3-3ζ and (c) *Cdkn1a*/p21 or (d) *Cdkn2c/*p18 was used to examine if knockdown of both proteins could restore differentiation, as determined by Oil Red-O staining (n=4 independent experiments). . Significant differences were assessed by one-way ANOVA and are indicated by *: p<0.05. Error bars represent S.E.M.

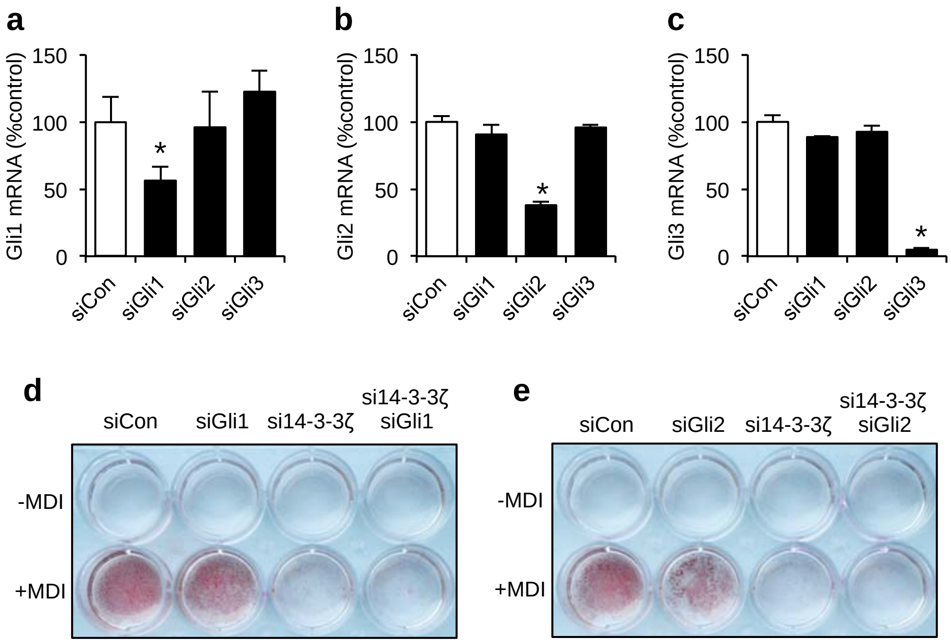

Supplementary Figure 7. Knockdown of Gli1 or Gli2 was unable to restore differentiation in 14-3-3ζ-depleted cells. (a-c) 3T3-L1 pre-adipocytes were transfected with siRNA against Gli1 (a), Gli2 (b), and Gli3 (c), followed by RNA isolation, and qPCR to measure transfection efficiencies (n=4 per group). **(d,e)** Co-transfection of siRNA against 14-3-3ζ and (d) Gli1 or (e) Gli2 was used to examine if knockdown of both proteins could restore differentiation, as determined by Oil Red-O staining (n=4 independent experiments). . Significant differences, as assessed by one-way ANOVA, are indicated by *: $p<0.05$. Error bars represent S.EM.

Fig. 1h

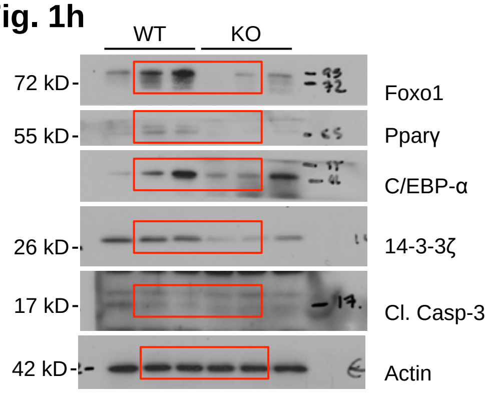

Fig. 2a

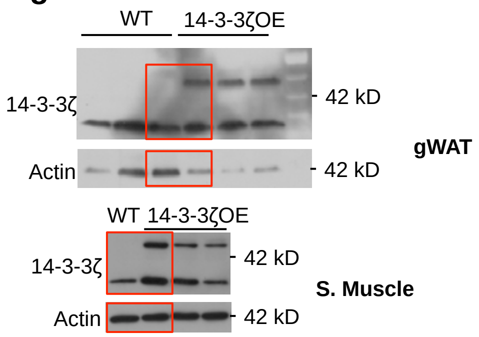

Fig. 3c

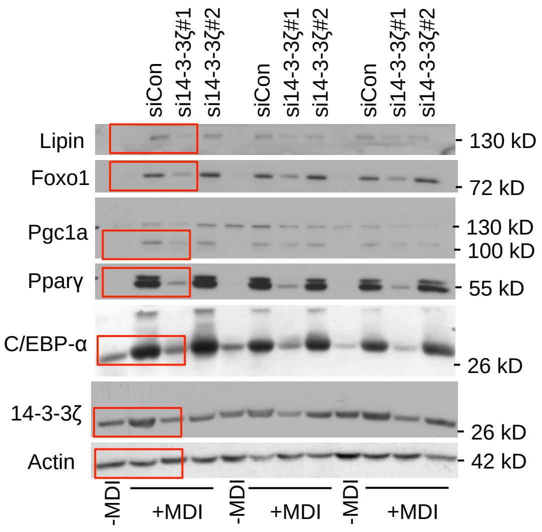

Fig. 3d

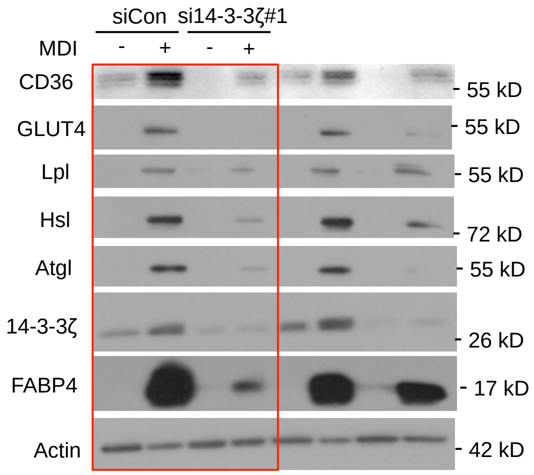

Supplementary Figure 8. Original Scans of Western Blots

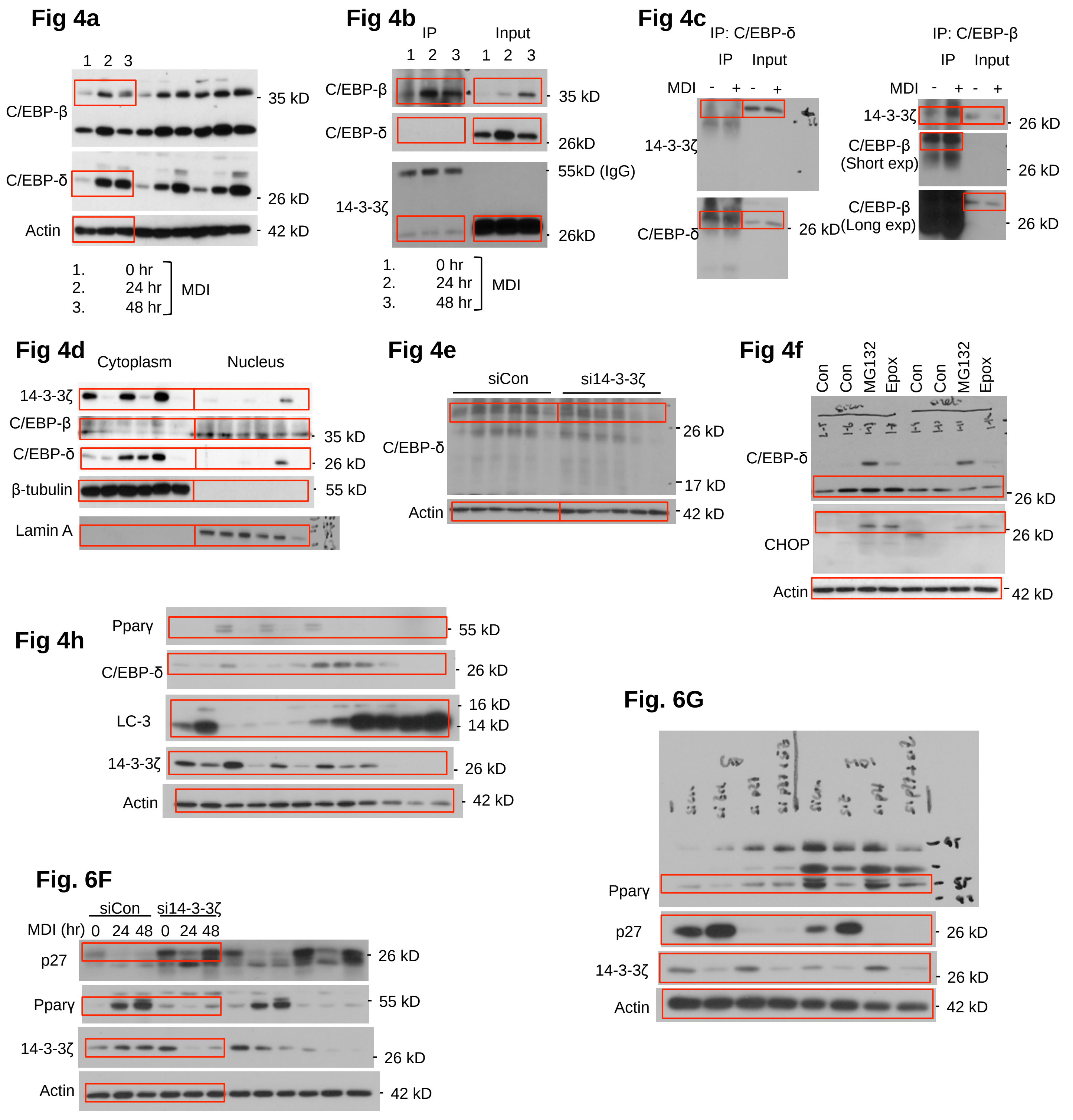

Supplementary Figure 8. Original Scans of Western Blots (Con)

Fig. 7e

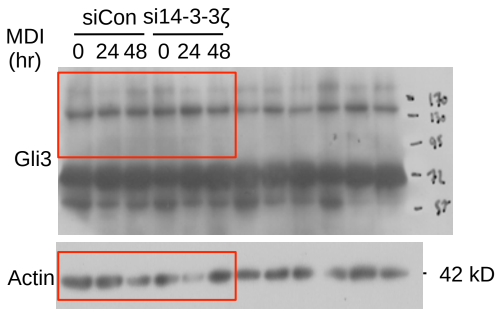

Fig. 7f

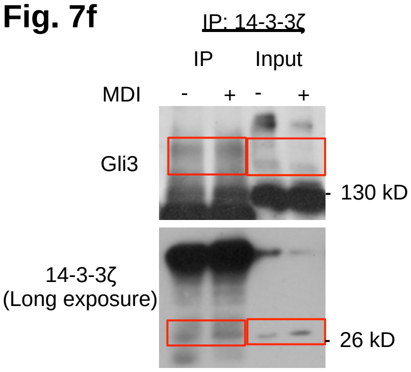

Fig. 7g

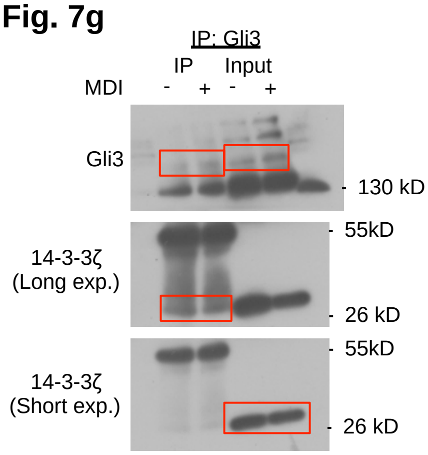

Fig. 7i

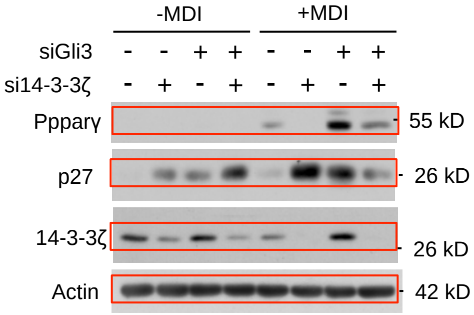

Supplementary Figure 1i

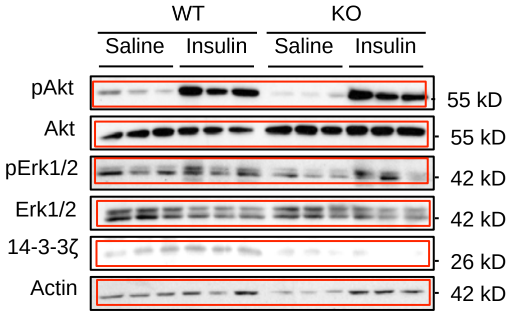

Supplementary Figure 8. Original Scans of Western Blots (Con)

Supplementary Table 1: Fasting plasma hormone concentrations in wild-type and 14-3-3ζKO mice

	WT	14-3-3ζKO
**Insulin** (ng/ ml)	0.34 ± 0.07	0.46 ± 0.06 *
**Total adiponectin** (μg/ ml)	4.08 ± 0.20	3.72 ± 0.21
**HMW adiponectin** (μg/ ml)	2.34 ± 0.12	2.11 ± 0.33
**Corticosterone** (ng/ ml)	337.01 ± 102.52	492.72 ± 106.03

n=5-7 per genotype
*: $p<0.05$ when compared to control

Supplementary Table 2: Top and bottom 25 most changed genes after 48 hours of differentiation relative to siCon-transfected cells

		siCon			siZeta		
		0	24	48	0	24	48
1	Krt15	1	103.110918	497.311385	2.66051696	106.477924	145.796555
2	Fam83a	1	46.9124964	368.012974	13.9652891	16.5395541	31.0906177
3	Krt13	1	127.748073	164.971274	2.10433604	81.8750183	45.9900747
4	2310014L17I	1	1.41740611	88.8011892	0.48230434	0.93882377	1.18106303
5	Nog	1	32.6942053	85.9266531	1.4719336	25.3915159	41.8079214
6	G0s2	1	8.61711945	84.4316184	0.35808763	1.04070388	5.65038824
7	Ren1	1	11.4951963	82.4971445	0	2.50361952	25.099235
8	Retn	1	9.39441789	63.502244	0.78129008	4.25782917	5.98152364
9	Klra17	1	0.25956822	45.3978432	2.8325008	0.18471283	13.8490692
10	Wnt4	1	15.9620244	45.3354564	0.76280073	24.8068077	45.1882186
11	Fam107a	1	3.09442691	44.2671316	0.47626856	6.86753891	89.1542819
12	Ramp3	1	135.193704	44.0992625	1.60991663	48.1753458	13.8392369
13	Cidec	1	6.49830578	40.2399565	0.44840159	1.500989	3.58758375
14	Arxes1	1	3.02212458	37.401903	0.9221424	5.72310422	8.75339344
16	Wfdc12	1	3.51105395	27.2384208	1.12254452	4.75851608	5.40723019
17	Sprr1b	1	89.5338408	23.7858685	0	27.95191	2.29093426
18	2210020M01	1	6.13623433	23.0792172	0.61979871	3.01962916	7.39391541
19	Sema3g	1	11.1457743	22.8030941	0.34652529	10.4947652	8.8418583
20	Gpd1	1	1.31666881	21.5113343	1.07285388	1.18917206	4.2092994
21	Rasd1	1	3.87338682	20.9617456	1.31982267	6.70150905	8.04514435
22	Ear2	1	8.46312165	20.706659	0	4.16784271	4.34382435
23	Cfd	1	1.50353483	20.5678042	0.7495344	0.23013684	1.74720513
24	Gm12347,Krt	1	23.300067	18.3841511	0.63432479	22.6639369	12.0704546
25	Mcam	1	5.38954757	18.2322709	2.55386429	3.34622458	5.3578282
9221	Apol9b	1	0.08373423	0.01948934	0.60923863	0.06082538	0.02648439
9222	Zbp1	1	0.0742234	0.01943856	0.41698993	0.02447823	0.0173708
9223	Oas3	1	0.07558769	0.01918292	0.47856067	0.01535832	0.01746165
9224	Ifi47,Olfr56	1	0.04992641	0.0163962	0.59134299	0.02718881	0.02831703
9225	BC023105	1	0.06714218	0.01464959	0.3617296	0.04433502	0.11591034
9226	Pde1b	1	0.02099799	0.01344245	1.13107782	0.02510594	0.00967196
9227	Gpr176	1	0.02035509	0.01135962	0.93039992	0.02116034	0.01790346
9228	Mmp13	1	0.02023406	0.01093638	0.27693908	0.00582188	0.00528267
9229	Serpinb9g	1	0.26502259	0.0107546	0.29989923	0.11016691	0.03022964
9230	Ces1a	1	0.05015896	0.01048362	0.81041207	0.04175486	0.01681023
9231	Vnn3	1	0.08024184	0.01027809	0.42577837	0.00669902	0.02750213
9232	Ubd	1	0.00266743	0.00997673	1.67059581	0.01285738	0.01558555
9233	Il1a	1	0.01260677	0.00964124	0.49442405	0.00619738	0.01258758
9234	Nov	1	0.03553023	0.00944395	1.42627053	0.12325194	0.14244752
9235	Rsad2	1	0.08694472	0.00941971	0.26700663	0.04735587	0.01091572
9236	Mamdc2	1	0.02190166	0.00847977	0.58208341	0.02056469	0.01430511
9237	Dlgap2	1	0.27744687	0.00742684	0.93054361	0.09900994	0.05647909
9238	Siglecg	1	0.07252997	0.00722883	0.93852424	0.02480858	0.0091241
9239	Gm12185,Tg	1	0.03304194	0.00669948	0.55308361	0.04808998	0.0012725
9240	Gm4070	1	0.02262252	0.00616123	0.31498816	0.00391003	0.00578352
9241	Mpeg1	1	0.03792581	0.00586986	0.25259452	0.01251313	0.00637423
9242	Mmp8	1	0.04044817	0.00553258	0.33967777	0.02070749	0.01046043
9243	I830012O16I	1	0.02556889	0.00407705	0.33324216	0.00908203	0.00616297
9244	Ifit3	1	0.03142822	0.00382907	0.31572258	0.01235294	0.00424276
9245	Tgtp2	1	0.01567832	0.0009063	0.3673668	0.02448039	0.00620549

Supplementary Table 3: Gene Set Enrichment Analysis for highly enriched genes in siCon-transfected 3T3-L1 cells at t=0 hr of differentiation

Term ID	Description	SIZE	ES	NES	NOM p-val	FDR q-val	FWER p-val	RANK AT MAX	LEADING EDGE
GO:0055114	oxidation-reduction process	542	0.19408107	5.144256	0	0	0	9295	tags=47%, list=28%, signal=64%
GO:0043207	response to external biotic stimulus	407	0.20895255	4.9130325	0	0	0	8659	tags=47%, list=26%, signal=62%
GO:0001817	regulation of cytokine production	371	0.22158052	4.882406	0	0	0	8667	tags=48%, list=26%, signal=64%
GO:0009607	response to biotic stimulus	419	0.20515685	4.811199	0	0	0	8659	tags=46%, list=26%, signal=62%
GO:0022603	regulation of anatomical structure morphogenesis	598	0.16492768	4.7478986	0	0	0	8626	tags=42%, list=26%, signal=56%
GO:0009611	response to wounding	353	0.21167463	4.528433	0	0	0	10556	tags=53%, list=32%, signal=76%
GO:0072521	purine-containing compound metabolic process	408	0.18785776	4.357321	0	0	0	8662	tags=45%, list=26%, signal=60%
GO:0044087	regulation of cellular component biogenesis	355	0.19560497	4.2718105	0	0	0	8551	tags=45%, list=26%, signal=60%
GO:0050776	regulation of immune response	342	0.19733883	4.2676053	0	0	0	11589	tags=54%, list=35%, signal=83%
GO:0043062	extracellular structure organization	144	0.28958005	4.116184	0	0	0	8654	tags=55%, list=26%, signal=74%
GO:0030198	extracellular matrix organization	143	0.28640112	4.0330143	0	0	0	8654	tags=55%, list=26%, signal=73%
GO:0008285	negative regulation of cell proliferation	468	0.16432111	3.9995213	0	0	0	8609	tags=42%, list=26%, signal=56%
GO:0016477	cell migration	520	0.15089844	3.8994722	0	0	0	9064	tags=42%, list=27%, signal=57%
GO:0001934	positive regulation of protein phosphorylation	497	0.15205948	3.8695254	0	0	0	8667	tags=41%, list=26%, signal=55%
GO:0043523	regulation of neuron apoptotic process	177	0.24906825	3.820211	0	0	0	8481	tags=50%, list=26%, signal=67%
GO:0010638	positive regulation of organelle organization	229	0.21925238	3.8053563	0	0	0	8586	tags=48%, list=26%, signal=64%
GO:1901137	carbohydrate derivative biosynthetic process	266	0.19304864	3.654203	0	0	0	8632	tags=45%, list=26%, signal=60%
GO:0030155	regulation of cell adhesion	256	0.19730611	3.6112642	0	0	0	8556	tags=45%, list=26%, signal=61%
GO:0050865	regulation of cell activation	335	0.16510855	3.5227149	0	0	0	8659	tags=42%, list=26%, signal=57%
GO:0040007	growth	311	0.16710086	3.4432998	0	0	0	8501	tags=42%, list=26%, signal=56%
GO:0050890	cognition	185	0.21452352	3.4257956	0	0	0	9082	tags=49%, list=27%, signal=67%
GO:0001775	cell activation	399	0.14664754	3.4140747	0	0	0	8682	tags=41%, list=26%, signal=54%
GO:0006605	protein targeting	183	0.21082048	3.329797	0	0	0	8654	tags=47%, list=26%, signal=63%
GO:0043900	regulation of multi-organism process	149	0.23285142	3.3284934	0	0	0	8359	tags=48%, list=25%, signal=64%
GO:0007610	behavior	462	0.13167499	3.3183427	0	0	0	10580	tags=45%, list=32%, signal=65%
GO:0048771	tissue remodeling	89	0.29241267	3.297901	0	0	0	8609	tags=55%, list=26%, signal=74%
GO:0019725	cellular homeostasis	394	0.14217415	3.294836	0	0	0	8662	tags=40%, list=26%, signal=54%
GO:0048609	multicellular organismal reproductive process	459	0.13473064	3.28107	0	0	0	9635	tags=42%, list=29%, signal=59%
GO:0051090	regulation of sequence-specific DNA binding transcription factor activity	274	0.17154701	3.2702584	0	0	0	9026	tags=44%, list=27%, signal=60%
GO:0072593	reactive oxygen species metabolic process	67	0.34582606	3.2687361	0	0	0	8871	tags=61%, list=27%, signal=83%
GO:0001816	cytokine production	95	0.27701637	3.1835132	0	0	0	7965	tags=52%, list=24%, signal=68%
GO:0034660	ncRNA metabolic process	129	0.2359121	3.1712277	0	0	0	8682	tags=50%, list=26%, signal=67%
GO:0014070	response to organic cyclic compound	407	0.13599451	3.1596463	0	0	0	8686	tags=40%, list=26%, signal=53%
GO:0048666	neuron development	512	0.12094986	3.1073432	0	1.31E-05	0.002	9223	tags=40%, list=28%, signal=54%
GO:0008283	cell proliferation	433	0.12619744	3.092265	0	1.30E-05	0.002	8682	tags=39%, list=26%, signal=52%
GO:0003014	renal system process	64	0.32456008	3.055742	0	1.26E-05	0.002	10010	tags=63%, list=30%, signal=89%
GO:0009636	response to toxic substance	79	0.28783017	3.0021021	0	1.19E-05	0.002	8550	tags=54%, list=26%, signal=73%
GO:0034470	ncRNA processing	91	0.26707628	2.93926	0	1.06E-05	0.002	8682	tags=53%, list=26%, signal=71%
GO:0007568	aging	87	0.26652527	2.9320092	0	1.05E-05	0.002	8359	tags=52%, list=25%, signal=69%
GO:1901615	organic hydroxy compound metabolic process	244	0.16002825	2.9219627	0	1.04E-05	0.002	9980	tags=46%, list=30%, signal=65%
GO:0006790	sulfur compound metabolic process	136	0.21240489	2.862802	0	2.47E-05	0.005	8613	tags=47%, list=26%, signal=63%
GO:0043161	proteasomal ubiquitin-dependent protein catabolic process	104	0.2336874	2.8272424	0	2.36E-05	0.005	8559	tags=49%, list=26%, signal=66%
GO:0001654	eye development	281	0.1430896	2.8267546	0	2.35E-05	0.005	9363	tags=42%, list=28%, signal=58%
GO:0010038	response to metal ion	155	0.19488443	2.824612	0	2.32E-05	0.005	8995	tags=46%, list=27%, signal=63%
GO:0072001	renal system development	187	0.17082882	2.7811756	0	3.49E-05	0.008	8576	tags=43%, list=26%, signal=57%
GO:0051186	cofactor metabolic process	184	0.17474271	2.765697	0	4.62E-05	0.01	8678	tags=43%, list=26%, signal=59%
GO:0006109	regulation of carbohydrate metabolic process	119	0.21456815	2.7228172	0	5.98E-05	0.014	9376	tags=50%, list=28%, signal=69%
GO:2000377	regulation of reactive oxygen species metabolic process	63	0.28893685	2.715942	0	6.29E-05	0.015	8355	tags=54%, list=25%, signal=72%
GO:0061458	reproductive system development	223	0.15969416	2.7109907	0	7.38E-05	0.018	8741	tags=42%, list=26%, signal=57%
GO:1901617	organic hydroxy compound biosynthetic process	90	0.24140081	2.691127	0	8.34E-05	0.021	10467	tags=56%, list=31%, signal=81%
GO:0043588	skin development	222	0.15162711	2.659821	0	1.17E-04	0.031	9370	tags=43%, list=28%, signal=60%
GO:0042157	lipoprotein metabolic process	59	0.285632	2.5989594	0	2.00E-04	0.056	8553	tags=54%, list=26%, signal=73%
GO:0007267	cell-cell signaling	475	0.10305165	2.5694382	0	2.45E-04	0.071	8662	tags=36%, list=26%, signal=48%
GO:0006732	coenzyme metabolic process	147	0.18224017	2.5620868	0.00198807	2.54E-04	0.075	8443	tags=44%, list=25%, signal=58%
GO:0030879	mammary gland development	97	0.21778959	2.4968085	0	3.55E-04	0.106	10604	tags=54%, list=32%, signal=78%
GO:0070646	protein modification by small protein removal	75	0.24913886	2.4934754	0	3.62E-04	0.109	8581	tags=51%, list=26%, signal=68%
GO:0021700	developmental maturation	172	0.16000745	2.4561114	0	4.56E-04	0.143	10172	tags=47%, list=31%, signal=67%
GO:0006091	generation of precursor metabolites and energy	132	0.1846152	2.4482758	0	4.87E-04	0.154	9251	tags=46%, list=28%, signal=64%
GO:0001503	ossification	142	0.17207217	2.400811	0.00193799	7.18E-04	0.226	8586	tags=43%, list=26%, signal=58%
GO:0009791	post-embryonic development	98	0.20367227	2.3751075	0	8.36E-04	0.262	7158	tags=42%, list=22%, signal=53%
GO:0071229	cellular response to acid	110	0.19269532	2.3599732	0	9.13E-04	0.284	19912	tags=79%, list=60%, signal=196%

Supplementary Table 4: Gene Set Enrichment Analysis for highly enriched genes in si14-3-3ζ-transfected 3T3-L1 cells at t=0 hr of differentiation

Term ID	Description	SIZE	ES	NES	NOM p-val	FDR q-val	FWER p-val	RANK AT MAX	LEADING EDGE
GO:0007049	cell cycle	528	-0.3056521	-7.94206	0	0	0	8955	tags=57%, list=27%, signal=77%
GO:0006259	DNA metabolic process	401	-0.2489749	-5.759177	0	0	0	8904	tags=51%, list=27%, signal=69%
GO:0018193	peptidyl-amino acid modification	410	-0.2426255	-5.5757174	0	0	0	8902	tags=51%, list=27%, signal=68%
GO:0007059	chromosome segregation	107	-0.4349217	-5.3092785	0	0	0	8893	tags=70%, list=27%, signal=95%
GO:0055086	nucleobase-containing small molecule metabolic process	515	-0.2022069	-5.2696376	0	0	0	8942	tags=47%, list=27%, signal=63%
GO:0016192	vesicle-mediated transport	489	-0.1954034	-4.900353	0	0	0	8967	tags=46%, list=27%, signal=62%
GO:0051338	regulation of transferase activity	502	-0.1830628	-4.857795	0	0	0	8777	tags=44%, list=26%, signal=59%
GO:0010608	posttranscriptional regulation of gene expression	312	-0.2196499	-4.4571986	0	0	0	9072	tags=49%, list=27%, signal=67%
GO:0016071	mRNA metabolic process	179	-0.2810754	-4.451542	0	0	0	8910	tags=55%, list=27%, signal=74%
GO:0043009	chordate embryonic development	565	-0.1618644	-4.417902	0	0	0	9188	tags=44%, list=28%, signal=59%
GO:0030334	regulation of cell migration	446	-0.1811758	-4.3825593	0	0	0	8894	tags=45%, list=27%, signal=60%
GO:0023014	signal transduction by phosphorylation	160	-0.2971594	-4.335813	0	0	0	8456	tags=55%, list=25%, signal=73%
GO:0006396	RNA processing	234	-0.2419045	-4.2817945	0	0	0	8924	tags=51%, list=27%, signal=69%
GO:0080135	regulation of cellular response to stress	306	-0.208088	-4.1767898	0	0	0	8902	tags=47%, list=27%, signal=64%
GO:0007167	enzyme linked receptor protein signaling pathway	372	-0.1805735	-4.079011	0	0	0	8902	tags=45%, list=27%, signal=60%
GO:0007507	heart development	375	-0.1787231	-4.0699983	0	0	0	8756	tags=44%, list=26%, signal=59%
GO:0010639	negative regulation of organelle organization	159	-0.2807691	-4.0543103	0	0	0	8904	tags=55%, list=27%, signal=74%
GO:0006508	proteolysis	341	-0.1930983	-4.023275	0	0	0	8858	tags=46%, list=27%, signal=62%
GO:0044772	mitotic cell cycle phase transition	85	-0.3715078	-3.988436	0	0	0	6848	tags=58%, list=21%, signal=72%
GO:0032886	regulation of microtubule-based process	110	-0.3330276	-3.9558992	0	0	0	8915	tags=60%, list=27%, signal=82%
GO:0007264	small GTPase mediated signal transduction	102	-0.3345701	-3.9269848	0	0	0	8144	tags=58%, list=24%, signal=76%
GO:0032259	methylation	135	-0.2895593	-3.9095492	0	0	0	8885	tags=56%, list=27%, signal=76%
GO:0061024	membrane organization	354	-0.1763437	-3.8309417	0	0	0	8947	tags=44%, list=27%, signal=60%
GO:0010563	negative regulation of phosphorus metabolic process	291	-0.1954303	-3.827398	0	0	0	8300	tags=44%, list=25%, signal=59%
GO:0051960	regulation of nervous system development	543	-0.1384103	-3.6872585	0	0	0	8946	tags=41%, list=27%, signal=55%
GO:0035295	tube development	417	-0.1529209	-3.675426	0	0	0	9094	tags=42%, list=27%, signal=58%
GO:0007163	establishment or maintenance of cell polarity	91	-0.3233953	-3.656385	0	0	0	6451	tags=52%, list=19%, signal=64%
GO:0040008	regulation of growth	461	-0.1448446	-3.5984418	0	0	0	8884	tags=41%, list=27%, signal=55%
GO:0032844	regulation of homeostatic process	282	-0.1829575	-3.5802863	0	0	0	9062	tags=45%, list=27%, signal=62%
GO:0016197	endosomal transport	101	-0.2962198	-3.4776554	0	0	0	8947	tags=56%, list=27%, signal=77%
GO:0034329	cell junction assembly	62	-0.3737445	-3.4713187	0	0	0	8513	tags=63%, list=26%, signal=84%
GO:0016311	dephosphorylation	185	-0.2183339	-3.466415	0	0	0	8958	tags=49%, list=27%, signal=66%
GO:0042176	regulation of protein catabolic process	170	-0.2244362	-3.4592838	0	0	0	8811	tags=49%, list=26%, signal=66%
GO:2001234	negative regulation of apoptotic signaling pathway	146	-0.2411573	-3.4360778	0	0	0	8871	tags=51%, list=27%, signal=69%
GO:0022610	biological adhesion	360	-0.1547073	-3.4122567	0	0	0	8675	tags=41%, list=26%, signal=55%
GO:0009125	nucleoside monophosphate catabolic process	173	-0.2177962	-3.3851202	0	0	0	8942	tags=49%, list=27%, signal=66%
GO:0009725	response to hormone stimulus	363	-0.1510749	-3.3512826	0	0	0	8956	tags=42%, list=27%, signal=57%
GO:0002009	morphogenesis of an epithelium	358	-0.1555922	-3.3489702	0	0	0	9094	tags=43%, list=27%, signal=58%
GO:0006260	DNA replication	85	-0.3097296	-3.3088357	0	0	0	8897	tags=58%, list=27%, signal=79%
GO:0006397	mRNA processing	118	-0.2591716	-3.3041995	0	0	0	8885	tags=53%, list=27%, signal=71%
GO:0001501	skeletal system development	338	-0.1541148	-3.2663224	0	0	0	9095	tags=43%, list=27%, signal=58%
GO:0005975	carbohydrate metabolic process	291	-0.1595047	-3.2057004	0	0	0	8570	tags=42%, list=26%, signal=56%
GO:0044723	single-organism carbohydrate metabolic process	279	-0.1594094	-3.0918698	0	0	0	8570	tags=42%, list=26%, signal=56%
GO:0044782	cilium organization	118	-0.2346532	-2.9884195	0	3.53E-06	0.001	8852	tags=50%, list=27%, signal=68%
GO:0030010	establishment of cell polarity	63	-0.3180787	-2.978343	0	3.50E-06	0.001	6862	tags=52%, list=21%, signal=66%
GO:0034330	cell junction organization	99	-0.2523095	-2.9613662	0	3.44E-06	0.001	8766	tags=52%, list=26%, signal=70%
GO:0061061	muscle structure development	339	-0.1393739	-2.9450989	0	3.41E-06	0.001	8950	tags=41%, list=27%, signal=55%
GO:0001655	urogenital system development	222	-0.1711532	-2.9394283	0	3.40E-06	0.001	7379	tags=39%, list=22%, signal=50%
GO:0042110	T cell activation	203	-0.1657692	-2.7623887	0	5.70E-05	0.019	11558	tags=51%, list=35%, signal=78%
GO:0007389	pattern specification process	373	-0.1253858	-2.7555656	0	5.68E-05	0.019	9518	tags=41%, list=29%, signal=57%
GO:0031669	cellular response to nutrient levels	77	-0.2605761	-2.7249963	0	6.44E-05	0.022	9062	tags=53%, list=27%, signal=73%
GO:0045444	fat cell differentiation	69	-0.2736957	-2.7130942	0	6.68E-05	0.023	7304	tags=49%, list=22%, signal=63%
GO:0040029	regulation of gene expression, epigenetic	126	-0.2059611	-2.7013478	0	7.26E-05	0.025	7957	tags=44%, list=24%, signal=58%
GO:0003002	regionalization	272	-0.1414191	-2.6830518	0	7.77E-05	0.026	9518	tags=43%, list=29%, signal=59%
GO:0050678	regulation of epithelial cell proliferation	221	-0.1583128	-2.6724598	0	7.96E-05	0.027	9517	tags=44%, list=29%, signal=62%

Supplementary Table 5: Gene Set Enrichment Analysis for highly enriched genes in siCon-transfected 3T3-L1 cells at t=24 hr of differentiation

Term ID	Description	SIZE	ES	NES	NOM p-val	FDR q-val	FWER p-val	RANK AT MAX	LEADING EDGE
GO:0040012	regulation of locomotion	497	0.21244763	5.387739	0	0	0	8094	tags=45%, list=24%, signal=59%
GO:0006396	RNA processing	234	0.29805347	5.2699904	0	0	0	8347	tags=55%, list=25%, signal=73%
GO:0055086	nucleobase-containing small molecule metabolic process	515	0.19650061	5.184911	0	0	0	8352	tags=44%, list=25%, signal=58%
GO:0016265	death	558	0.18157847	4.9461055	0	0	0	8424	tags=43%, list=25%, signal=57%
GO:0034660	ncRNA metabolic process	129	0.34719357	4.5371804	0	0	0	8347	tags=60%, list=25%, signal=79%
GO:0001817	regulation of cytokine production	371	0.19957583	4.492846	0	0	0	8315	tags=45%, list=25%, signal=59%
GO:0044087	regulation of cellular component biogenesis	355	0.20390359	4.373157	0	0	0	8278	tags=45%, list=25%, signal=59%
GO:0061061	muscle structure development	339	0.20603824	4.2976737	0	0	0	9108	tags=48%, list=27%, signal=65%
GO:0034470	ncRNA processing	91	0.37635103	4.2853575	0	0	0	8347	tags=63%, list=25%, signal=83%
GO:0022603	regulation of anatomical structure morphogenesis	598	0.14927629	4.2661633	0	0	0	8303	tags=40%, list=25%, signal=52%
GO:0043066	negative regulation of apoptotic process	529	0.16155359	4.224526	0	0	0	8227	tags=41%, list=25%, signal=53%
GO:0009611	response to wounding	353	0.19134147	4.1789083	0	0	0	7928	tags=43%, list=24%, signal=56%
GO:0050776	regulation of immune response	342	0.19050147	4.049902	0	0	0	9675	tags=48%, list=29%, signal=67%
GO:0050865	regulation of cell activation	335	0.18179357	3.973754	0	0	0	7812	tags=41%, list=23%, signal=54%
GO:0043062	extracellular structure organization	144	0.2789935	3.9685378	0	0	0	7619	tags=51%, list=23%, signal=65%
GO:0001934	positive regulation of protein phosphorylation	497	0.15667813	3.965975	0	0	0	8315	tags=40%, list=25%, signal=53%
GO:0030198	extracellular matrix organization	143	0.28254545	3.9466043	0	0	0	7619	tags=51%, list=23%, signal=66%
GO:0008283	cell proliferation	433	0.16311306	3.9462755	0	0	0	8392	tags=41%, list=25%, signal=55%
GO:0001501	skeletal system development	338	0.17928271	3.9216406	0	0	0	8363	tags=43%, list=25%, signal=57%
GO:0010638	positive regulation of organelle organization	229	0.21344368	3.7548091	0	0	0	8197	tags=46%, list=25%, signal=60%
GO:0005975	carbohydrate metabolic process	291	0.18287592	3.656827	0	0	0	8369	tags=43%, list=25%, signal=57%
GO:0001701	in utero embryonic development	360	0.16367608	3.6343331	0	0	0	8378	tags=41%, list=25%, signal=55%
GO:0007517	muscle organ development	194	0.2262331	3.6268895	0	0	0	10346	tags=54%, list=31%, signal=77%
GO:0043900	regulation of multi-organism process	149	0.25343877	3.5959451	0	0	0	8347	tags=50%, list=25%, signal=67%
GO:0044723	single-organism carbohydrate metabolic process	279	0.18351737	3.5594027	0	0	0	8369	tags=43%, list=25%, signal=57%
GO:0052548	regulation of endopeptidase activity	262	0.18440472	3.5022814	0	0	0	8257	tags=43%, list=25%, signal=57%
GO:1901137	carbohydrate derivative biosynthetic process	266	0.18586287	3.5001419	0	0	0	8369	tags=44%, list=25%, signal=58%
GO:0048878	chemical homeostasis	581	0.1271358	3.4640484	0	0	0	8322	tags=38%, list=25%, signal=49%
GO:0014070	response to organic cyclic compound	407	0.14382826	3.4259026	0	0	0	8347	tags=39%, list=25%, signal=52%
GO:0007167	enzyme linked receptor protein signaling pathway	372	0.15466899	3.4235928	0	0	0	7964	tags=39%, list=24%, signal=51%
GO:0043207	response to external biotic stimulus	407	0.14570372	3.3971062	0	0	0	8122	tags=39%, list=24%, signal=51%
GO:0009607	response to biotic stimulus	419	0.1441654	3.3847375	0	0	0	8122	tags=39%, list=24%, signal=51%
GO:0010038	response to metal ion	155	0.23025984	3.3802621	0	0	0	10184	tags=54%, list=31%, signal=77%
GO:0060627	regulation of vesicle-mediated transport	247	0.18369074	3.3723195	0	0	0	8341	tags=43%, list=25%, signal=57%
GO:0001816	cytokine production	95	0.29122534	3.3719344	0	0	0	9594	tags=58%, list=29%, signal=81%
GO:0007155	cell adhesion	353	0.15152316	3.3673992	0	0	0	8296	tags=40%, list=25%, signal=53%
GO:0014706	striated muscle tissue development	240	0.18162338	3.3227522	0	0	0	8551	tags=44%, list=26%, signal=58%
GO:0001775	cell activation	399	0.1416936	3.3109164	0	0	0	7428	tags=36%, list=22%, signal=46%
GO:0001503	ossification	142	0.22945707	3.2641237	0	0	0	8091	tags=47%, list=24%, signal=62%
GO:0022610	biological adhesion	360	0.14651184	3.2299366	0	0	0	8296	tags=39%, list=25%, signal=52%
GO:0006732	coenzyme metabolic process	147	0.2231046	3.1844828	0	0	0	7995	tags=46%, list=24%, signal=61%
GO:0048660	regulation of smooth muscle cell proliferation	89	0.2832502	3.1780713	0	0	0	7792	tags=52%, list=23%, signal=67%
GO:0051186	cofactor metabolic process	184	0.19613604	3.1538174	0	0	0	8332	tags=45%, list=25%, signal=59%
GO:1901566	organonitrogen compound biosynthetic process	290	0.1602083	3.1329267	0	6.40E-06	0.001	8363	tags=41%, list=25%, signal=54%
GO:0040007	growth	311	0.15136005	3.1053603	0	6.25E-06	0.001	8378	tags=40%, list=25%, signal=53%
GO:0003006	developmental process involved in reproduction	382	0.13212433	2.9544032	0	2.20E-05	0.004	8452	tags=38%, list=25%, signal=51%
GO:0071826	ribonucleoprotein complex subunit organization	84	0.27344772	2.938143	0	2.16E-05	0.004	8347	tags=52%, list=25%, signal=70%
GO:0001655	urogenital system development	222	0.16573142	2.8966348	0	2.06E-05	0.004	8305	tags=41%, list=25%, signal=55%
GO:0061458	reproductive system development	223	0.16713199	2.8827765	0	3.05E-05	0.006	8048	tags=41%, list=24%, signal=53%
GO:0045444	fat cell differentiation	69	0.2955547	2.8734424	0	3.02E-05	0.006	4649	tags=43%, list=14%, signal=50%
GO:0022411	cellular component disassembly	73	0.28149635	2.8613145	0	3.97E-05	0.008	8424	tags=53%, list=25%, signal=71%
GO:0048863	stem cell differentiation	238	0.15504247	2.7845647	0	6.54E-05	0.014	8293	tags=40%, list=25%, signal=53%
GO:0015931	nucleobase-containing compound transport	62	0.30122462	2.778344	0	6.95E-05	0.015	7700	tags=53%, list=23%, signal=69%
GO:0060348	bone development	114	0.22394072	2.764853	0	7.25E-05	0.016	8329	tags=47%, list=25%, signal=63%
GO:0006790	sulfur compound metabolic process	136	0.19975239	2.731951	0	8.86E-05	0.02	8543	tags=46%, list=26%, signal=61%
GO:0010498	proteasomal protein catabolic process	110	0.22406119	2.7018383	0	1.05E-04	0.025	7990	tags=46%, list=24%, signal=61%
GO:0008015	blood circulation	232	0.1518458	2.6832983	0	1.22E-04	0.03	4588	tags=29%, list=14%, signal=33%
GO:0048589	developmental growth	182	0.1720337	2.6730757	0	1.27E-04	0.032	8378	tags=42%, list=25%, signal=56%
GO:0006275	regulation of DNA replication	92	0.23780353	2.6515293	0	1.60E-04	0.04	8378	tags=49%, list=25%, signal=65%
GO:0021700	developmental maturation	172	0.16876563	2.631705	0	1.87E-04	0.048	8529	tags=42%, list=26%, signal=57%
GO:0072001	renal system development	187	0.16289128	2.6150043	0	1.98E-04	0.052	8305	tags=41%, list=25%, signal=55%
GO:0006091	generation of precursor metabolites and energy	132	0.19427703	2.6053846	0	2.00E-04	0.053	8931	tags=46%, list=27%, signal=63%
GO:0007568	aging	87	0.23607044	2.589664	0	2.22E-04	0.06	7458	tags=46%, list=22%, signal=59%
GO:0048864	stem cell development	182	0.1635545	2.5591557	0	2.94E-04	0.083	8293	tags=41%, list=25%, signal=55%
GO:0001890	placenta development	134	0.18513672	2.5143604	0	3.49E-04	0.104	7764	tags=42%, list=23%, signal=54%
GO:0007610	behavior	462	0.10307307	2.5114343	0	3.63E-04	0.108	8351	tags=35%, list=25%, signal=46%
GO:0043467	regulation of generation of precursor metabolites and energy	63	0.26253107	2.492855	0	3.97E-04	0.121	10287	tags=57%, list=31%, signal=83%

Supplementary Table 6: Gene Set Enrichment Analysis for highly enriched genes in si14-3-3ζ-transfected 3T3-L1 cells at t=24 hr of differentiation

Term ID	Description	SIZE	ES	NES	NOM p-val	FDR q-val	FWER p-val	RANK AT MAX	LEADING EDGE
GO:0007049	cell cycle	528	-0.2831359	-7.4872613	0	0	0	9188	tags=55%, list=28%, signal=75%
GO:0007017	microtubule-based process	310	-0.3144054	-6.467956	0	0	0	9166	tags=59%, list=28%, signal=80%
GO:0019236	response to pheromone	81	-0.6070486	-6.373813	0	0	0	11064	tags=94%, list=33%, signal=140%
GO:0032990	cell part morphogenesis	432	-0.2505095	-5.991905	0	0	0	9175	tags=52%, list=28%, signal=71%
GO:0006508	proteolysis	432	-0.2505095	-5.991905	0	0	0	9175	tags=52%, list=28%, signal=71%
GO:0044782	cilium organization	118	-0.4375238	-5.7203217	0	0	0	9175	tags=71%, list=28%, signal=98%
GO:0019439	aromatic compound catabolic process	459	-0.2322176	-5.674294	0	0	0	9193	tags=51%, list=28%, signal=69%
GO:0006259	DNA metabolic process	401	-0.2427018	-5.6556363	0	0	0	9193	tags=52%, list=28%, signal=70%
GO:0006325	chromatin organization	309	-0.2697313	-5.4739413	0	0	0	9194	tags=54%, list=28%, signal=74%
GO:0051301	cell division	177	-0.3343362	-5.2775636	0	0	0	8105	tags=58%, list=24%, signal=76%
GO:1901657	glycosyl compound metabolic process	364	-0.2324466	-5.071288	0	0	0	9165	tags=51%, list=28%, signal=69%
GO:0016192	vesicle-mediated transport	489	-0.1946403	-5.0183825	0	0	0	9196	tags=47%, list=28%, signal=64%
GO:0018193	peptidyl-amino acid modification	410	-0.2175443	-4.9818916	0	0	0	9158	tags=49%, list=28%, signal=67%
GO:0051338	regulation of transferase activity	502	-0.194784	-4.980715	0	0	0	9188	tags=47%, list=28%, signal=64%
GO:0072521	purine-containing compound metabolic process	408	-0.2048624	-4.8410153	0	0	0	9165	tags=48%, list=28%, signal=65%
GO:0007059	chromosome segregation	107	-0.382943	-4.7907443	0	0	0	9062	tags=65%, list=27%, signal=90%
GO:0080135	regulation of cellular response to stress	306	-0.2170521	-4.579681	0	0	0	9150	tags=49%, list=28%, signal=67%
GO:0030029	actin filament-based process	273	-0.2200338	-4.283411	0	0	0	9066	tags=49%, list=27%, signal=67%
GO:0009314	response to radiation	265	-0.2234595	-4.1502066	0	0	0	9193	tags=50%, list=28%, signal=68%
GO:0043009	chordate embryonic development	565	-0.1529663	-4.1352863	0	0	0	9420	tags=43%, list=28%, signal=59%
GO:0010608	posttranscriptional regulation of gene expression	312	-0.1975655	-4.064476	0	0	0	9160	tags=47%, list=28%, signal=64%
GO:0032886	regulation of microtubule-based process	110	-0.3247903	-4.0476723	0	0	0	9188	tags=60%, list=28%, signal=83%
GO:0035295	tube development	417	-0.1643281	-4.003794	0	0	0	8241	tags=41%, list=25%, signal=54%
GO:0051052	regulation of DNA metabolic process	226	-0.2308524	-3.9971397	0	0	0	9150	tags=50%, list=28%, signal=69%
GO:0043065	positive regulation of apoptotic process	325	-0.1887853	-3.935051	0	0	0	8825	tags=45%, list=27%, signal=61%
GO:0016311	dephosphorylation	185	-0.2495791	-3.9343326	0	0	0	9183	tags=52%, list=28%, signal=72%
GO:0009125	nucleoside monophosphate catabolic process	173	-0.2517288	-3.8621404	0	0	0	9165	tags=53%, list=28%, signal=72%
GO:0000910	cytokinesis	62	-0.4175927	-3.8414881	0	0	0	7594	tags=65%, list=23%, signal=83%
GO:0040008	regulation of growth	461	-0.1533582	-3.7960684	0	0	0	9254	tags=43%, list=28%, signal=59%
GO:0016071	mRNA metabolic process	179	-0.240148	-3.7637007	0	0	0	9149	tags=51%, list=28%, signal=71%
GO:0019953	sexual reproduction	447	-0.1479521	-3.7386298	0	0	0	9207	tags=42%, list=28%, signal=58%
GO:0046822	regulation of nucleocytoplasmic transport	156	-0.253061	-3.7195182	0	0	0	8678	tags=51%, list=26%, signal=69%
GO:0061024	membrane organization	354	-0.1672895	-3.6894257	0	0	0	9057	tags=44%, list=27%, signal=60%
GO:0001944	vasculature development	366	-0.1664309	-3.6275527	0	0	0	9155	tags=44%, list=28%, signal=60%
GO:1901615	organic hydroxy compound metabolic process	244	-0.2007189	-3.62659	0	0	0	9184	tags=48%, list=28%, signal=65%
GO:0007264	small GTPase mediated signal transduction	102	-0.3139367	-3.6119018	0	0	0	8502	tags=57%, list=26%, signal=76%
GO:0007389	pattern specification process	373	-0.1607804	-3.5985134	0	0	0	8800	tags=42%, list=26%, signal=57%
GO:0051960	regulation of nervous system development	543	-0.1272674	-3.5028615	0	0	0	9188	tags=40%, list=28%, signal=55%
GO:0016485	protein processing	148	-0.243022	-3.4760413	0	0	0	8582	tags=50%, list=26%, signal=67%
GO:0032259	methylation	135	-0.247974	-3.437004	0	0	0	8538	tags=50%, list=26%, signal=67%
GO:0009123	nucleoside monophosphate metabolic process	221	-0.1963004	-3.4164302	0	0	0	9165	tags=47%, list=28%, signal=65%
GO:0044772	mitotic cell cycle phase transition	85	-0.3172732	-3.404948	0	0	0	9038	tags=59%, list=27%, signal=81%
GO:0051604	protein maturation	160	-0.2291241	-3.399167	0	0	0	8837	tags=49%, list=27%, signal=67%
GO:0060249	anatomical structure homeostasis	162	-0.2291472	-3.3692312	0	0	0	8634	tags=49%, list=26%, signal=66%
GO:0007163	establishment or maintenance of cell polarity	91	-0.2992168	-3.3517835	0	0	0	8349	tags=55%, list=25%, signal=73%
GO:0003002	regionalization	272	-0.1703381	-3.220084	0	3.93E-06	0.001	9420	tags=45%, list=28%, signal=63%
GO:0048736	appendage development	144	-0.2316308	-3.2036314	0	3.88E-06	0.001	9420	tags=51%, list=28%, signal=71%
GO:0010565	regulation of cellular ketone metabolic process	99	-0.271486	-3.17534	0	3.76E-06	0.001	8802	tags=54%, list=26%, signal=73%
GO:0030101	natural killer cell activation	52	-0.3578313	-3.1015046	0	3.62E-06	0.001	13060	tags=75%, list=39%, signal=123%
GO:0045862	positive regulation of proteolysis	76	-0.2914044	-3.0537364	0	3.55E-06	0.001	9147	tags=57%, list=28%, signal=78%
GO:0023014	signal transduction by phosphorylation	160	-0.2075955	-3.049023	0	3.52E-06	0.001	7679	tags=44%, list=23%, signal=57%
GO:0035303	regulation of dephosphorylation	104	-0.2437877	-2.927068	0	1.01E-05	0.003	7267	tags=46%, list=22%, signal=59%
GO:0007626	locomotory behavior	181	-0.1841021	-2.9213166	0	1.01E-05	0.003	8977	tags=45%, list=27%, signal=62%
GO:0031647	regulation of protein stability	104	-0.2456094	-2.8903446	0	1.32E-05	0.004	9125	tags=52%, list=27%, signal=71%
GO:0007224	smoothened signaling pathway	60	-0.3212461	-2.8862612	0	1.32E-05	0.004	8181	tags=57%, list=25%, signal=75%
GO:0043330	response to exogenous dsRNA	51	-0.3332731	-2.780746	0	3.60E-05	0.012	13060	tags=73%, list=39%, signal=119%
GO:0008380	RNA splicing	85	-0.2540477	-2.7517698	0	4.74E-05	0.016	9179	tags=53%, list=28%, signal=73%
GO:0048489	synaptic vesicle transport	58	-0.3102233	-2.745005	0	4.94E-05	0.017	9196	tags=59%, list=28%, signal=81%
GO:0045216	cell-cell junction organization	90	-0.2397021	-2.7281938	0	5.65E-05	0.02	8678	tags=50%, list=26%, signal=67%
GO:0042157	lipoprotein metabolic process	59	-0.3015145	-2.6963456	0	6.63E-05	0.024	9155	tags=58%, list=28%, signal=79%
GO:0030010	establishment of cell polarity	63	-0.2891778	-2.666217	0.00194553	8.35E-05	0.03	8349	tags=54%, list=25%, signal=72%

Supplementary Table 7: Gene Set Enrichment Analysis for highly enriched genes in siCon-transfected 3T3-L1 cells at t=48 hr of differentiation

Term ID	Description	SIZE	ES	NES	NOM p-val	FDR q-val	FWER p-val	RANK AT MAX	LEADING EDGE
GO:0006396	RNA processing	234	0.45348072	8.155624	0	0	0	8615	tags=71%, list=26%, signal=95%
GO:0044711	single-organism biosynthetic process	593	0.25341666	7.161537	0	0	0	8601	tags=51%, list=26%, signal=67%
GO:0006259	DNA metabolic process	401	0.29146734	6.7521944	0	0	0	8667	tags=55%, list=26%, signal=73%
GO:0007049	cell cycle	528	0.24697402	6.639975	0	0	0	8606	tags=50%, list=26%, signal=67%
GO:0006082	organic acid metabolic process	545	0.24974717	6.6135774	0	0	0	8365	tags=50%, list=25%, signal=65%
GO:0022402	cell cycle process	507	0.24789675	6.559803	0	0	0	8606	tags=50%, list=26%, signal=67%
GO:0055086	nucleobase-containing small molecule metabolic process	515	0.24806337	6.450278	0	0	0	8601	tags=50%, list=26%, signal=67%
GO:0022613	ribonucleoprotein complex biogenesis	138	0.4674655	6.3135686	0	0	0	8615	tags=72%, list=26%, signal=97%
GO:0034660	ncRNA metabolic process	129	0.47945255	6.288133	0	0	0	8606	tags=74%, list=26%, signal=99%
GO:0070271	protein complex biogenesis	538	0.23394142	6.2283325	0	0	0	8601	tags=49%, list=26%, signal=65%
GO:0006412	translation	110	0.4955612	6.019969	0	0	0	10479	tags=81%, list=32%, signal=118%
GO:0034470	ncRNA processing	91	0.53382814	5.9275823	0	0	0	8606	tags=79%, list=26%, signal=106%
GO:0051276	chromosome organization	452	0.23955186	5.8865075	0	0	0	8547	tags=49%, list=26%, signal=66%
GO:0055114	oxidation-reduction process	542	0.21895446	5.822046	0	0	0	9463	tags=50%, list=28%, signal=69%
GO:0034654	nucleobase-containing compound biosynthetic process	599	0.196765	5.4992146	0	0	0	8951	tags=46%, list=27%, signal=62%
GO:0016071	mRNA metabolic process	179	0.340486	5.3588963	0	0	0	8615	tags=60%, list=26%, signal=80%
GO:0006281	DNA repair	194	0.3359924	5.3560553	0	0	0	8603	tags=59%, list=26%, signal=80%
GO:0016482	cytoplasmic transport	341	0.24457608	5.32071	0	0	0	8527	tags=50%, list=26%, signal=66%
GO:0034655	nucleobase-containing compound catabolic process	430	0.22035122	5.186867	0	0	0	8542	tags=47%, list=26%, signal=63%
GO:0018193	peptidyl-amino acid modification	410	0.21663947	5.109822	0	0	0	8618	tags=47%, list=26%, signal=63%
GO:0044265	cellular macromolecule catabolic process	370	0.22094665	4.9241476	0	0	0	8551	tags=48%, list=26%, signal=63%
GO:0072521	purine-containing compound metabolic process	345	0.22279495	4.90054	0	0	0	8667	tags=48%, list=26%, signal=64%
GO:0007059	chromosome segregation	107	0.39467025	4.8922157	0	0	0	7739	tags=63%, list=23%, signal=81%
GO:0009123	nucleoside monophosphate metabolic process	221	0.27911544	4.836389	0	0	0	8534	tags=53%, list=26%, signal=71%
GO:0006732	coenzyme metabolic process	147	0.34142753	4.814901	0	0	0	8602	tags=60%, list=26%, signal=80%
GO:0090407	organophosphate biosynthetic process	214	0.28098145	4.785614	0	0	0	8585	tags=54%, list=26%, signal=72%
GO:0009167	purine ribonucleoside monophosphate metabolic process	207	0.2764456	4.67177	0	0	0	8534	tags=53%, list=26%, signal=71%
GO:1901566	organonitrogen compound biosynthetic process	290	0.22757407	4.6622267	0	0	0	10041	tags=53%, list=30%, signal=75%
GO:1901657	glycosyl compound metabolic process	56	0.52863514	4.653685	0	0	0	8577	tags=79%, list=26%, signal=106%
GO:0045333	cellular respiration	56	0.52863514	4.653685	0	0	0	8577	tags=79%, list=26%, signal=106%
GO:0032787	monocarboxylic acid metabolic process	261	0.2494226	4.645359	0	0	0	8460	tags=50%, list=25%, signal=67%
GO:0051186	cofactor metabolic process	184	0.29756078	4.6320844	0	0	0	8231	tags=54%, list=25%, signal=72%
GO:0010675	regulation of cellular carbohydrate metabolic process	114	0.35968408	4.5526285	0	0	0	8206	tags=61%, list=25%, signal=80%
GO:0006109	regulation of carbohydrate metabolic process	119	0.3510829	4.507318	0	0	0	8206	tags=60%, list=25%, signal=79%
GO:0008380	RNA splicing	85	0.41253054	4.4456234	0	0	0	8615	tags=67%, list=26%, signal=90%
GO:0010608	posttranscriptional regulation of gene expression	312	0.22042993	4.369343	0	0	0	8618	tags=48%, list=26%, signal=64%
GO:0000122	negative regulation of transcription from RNA polymerase II promoter	523	0.16745126	4.3549905	0	0	0	8951	tags=43%, list=27%, signal=58%
GO:1901605	alpha-amino acid metabolic process	128	0.32277107	4.346789	0	0	0	7752	tags=55%, list=23%, signal=72%
GO:0009165	nucleotide biosynthetic process	115	0.3352763	4.281629	0	0	0	8551	tags=59%, list=26%, signal=79%
GO:0006091	generation of precursor metabolites and energy	132	0.32394874	4.252611	0	0	0	8163	tags=57%, list=25%, signal=75%
GO:0071103	DNA conformation change	91	0.37958997	4.2215014	0	0	0	8605	tags=64%, list=26%, signal=86%
GO:0019216	regulation of lipid metabolic process	215	0.2460069	4.182391	0	0	0	8575	tags=50%, list=26%, signal=67%
GO:0000278	mitotic cell cycle	213	0.24448359	4.1124587	0	0	0	7845	tags=48%, list=24%, signal=62%
GO:0006417	regulation of translation	148	0.28977555	4.1067066	0	0	0	8605	tags=55%, list=26%, signal=74%
GO:0071826	ribonucleoprotein complex subunit organization	84	0.37278187	4.066121	0	0	0	8615	tags=63%, list=26%, signal=85%
GO:0043066	negative regulation of apoptotic process	529	0.15085775	4.03474	0	0	0	8577	tags=41%, list=26%, signal=54%
GO:0006260	DNA replication	85	0.36565262	3.9686887	0	0	0	8605	tags=62%, list=26%, signal=84%
GO:0022618	ribonucleoprotein complex assembly	78	0.3828114	3.967152	0	0	0	8615	tags=64%, list=26%, signal=86%
GO:0043648	dicarboxylic acid metabolic process	58	0.43171698	3.962866	0	0	0	8601	tags=69%, list=26%, signal=93%
GO:0008610	lipid biosynthetic process	250	0.2142302	3.9301527	0	0	0	9954	tags=51%, list=30%, signal=73%
GO:0043467	regulation of generation of precursor metabolites and energy	63	0.41324654	3.8752625	0	0	0	10563	tags=73%, list=32%, signal=107%
GO:0009125	nucleoside monophosphate catabolic process	173	0.25343305	3.8738637	0	0	0	8530	tags=51%, list=26%, signal=68%
GO:0009128	purine nucleoside monophosphate catabolic process	171	0.25351968	3.7953331	0	0	0	8530	tags=51%, list=26%, signal=68%
GO:1901137	carbohydrate derivative biosynthetic process	266	0.19550423	3.77726	0	0	0	8551	tags=45%, list=26%, signal=60%
GO:0007346	regulation of mitotic cell cycle	263	0.19745618	3.728459	0	0	0	8910	tags=46%, list=27%, signal=63%
GO:0006979	response to oxidative stress	210	0.21380582	3.6984947	0	0	0	7977	tags=45%, list=24%, signal=59%
GO:0048878	chemical homeostasis	581	0.13481756	3.6839356	0	0	0	9788	tags=43%, list=29%, signal=59%
GO:0072522	purine-containing compound biosynthetic process	81	0.34709257	3.654688	0	0	0	8601	tags=60%, list=26%, signal=81%
GO:0008283	cell proliferation	433	0.14874993	3.6115208	0	0	0	8633	tags=41%, list=26%, signal=54%
GO:0051289	protein homotetramerization	53	0.42122546	3.5926993	0	0	0	8601	tags=68%, list=26%, signal=91%
GO:0090305	nucleic acid phosphodiester bond hydrolysis	111	0.28984106	3.5713568	0	0	0	8667	tags=55%, list=26%, signal=74%
GO:0050684	regulation of mRNA processing	56	0.41110593	3.5584314	0	0	0	8322	tags=66%, list=25%, signal=88%
GO:0046395	carboxylic acid catabolic process	118	0.2788492	3.5279171	0	0	0	8231	tags=53%, list=25%, signal=70%
GO:0051301	cell division	177	0.22356687	3.50205	0	0	0	8573	tags=48%, list=26%, signal=64%
GO:0072594	establishment of protein localization to organelle	138	0.25182572	3.4979937	0	0	0	8527	tags=51%, list=26%, signal=68%
GO:0040007	growth	311	0.17040493	3.4962597	0	0	0	8606	tags=43%, list=26%, signal=57%
GO:0061061	muscle structure development	339	0.1625581	3.492189	0	0	0	9362	tags=44%, list=28%, signal=61%
GO:0010565	regulation of cellular ketone metabolic process	99	0.29853508	3.4867072	0	0	0	8575	tags=56%, list=26%, signal=75%
GO:0006790	sulfur compound metabolic process	136	0.24942626	3.4664757	0	0	0	8365	tags=50%, list=25%, signal=67%
GO:0030258	lipid modification	97	0.29885152	3.43641	0	0	0	8602	tags=56%, list=26%, signal=75%
GO:0015931	nucleobase-containing compound transport	62	0.35710326	3.3229434	0	0	0	8527	tags=61%, list=26%, signal=82%
GO:0006302	double-strand break repair	83	0.31116983	3.31057	0	0	0	7706	tags=54%, list=23%, signal=70%
GO:0050727	regulation of inflammatory response	192	0.20188485	3.2793875	0	0	0	8392	tags=45%, list=25%, signal=60%
GO:0045444	fat cell differentiation	69	0.32003817	3.2237744	0	0	0	1427	tags=36%, list=4%, signal=38%
GO:0006310	DNA recombination	90	0.2875259	3.2177663	0	0	0	7829	tags=52%, list=24%, signal=68%
GO:0031331	positive regulation of cellular catabolic process	109	0.25795695	3.2112093	0	0	0	7923	tags=50%, list=24%, signal=65%
GO:0046890	regulation of lipid biosynthetic process	110	0.2543825	3.1788762	0	5.22E-06	0.001	10008	tags=55%, list=30%, signal=79%
GO:0051050	positive regulation of transport	571	0.11245929	3.1780813	0	5.19E-06	0.001	8553	tags=37%, list=26%, signal=49%
GO:0032259	methylation	135	0.23327997	3.1504214	0	5.12E-06	0.001	8530	tags=49%, list=26%, signal=65%
GO:0017038	protein import	97	0.2700953	3.1372437	0	4.99E-06	0.001	8527	tags=53%, list=26%, signal=71%
GO:0006575	cellular modified amino acid metabolic process	123	0.24457459	3.1208131	0	4.96E-06	0.001	8928	tags=51%, list=27%, signal=70%
GO:0044723	single-organism carbohydrate metabolic process	279	0.15623568	3.107897	0	4.90E-06	0.001	7600	tags=38%, list=23%, signal=49%
GO:0007126	meiosis	96	0.2688616	3.0972157	0	4.87E-06	0.001	8057	tags=51%, list=24%, signal=67%
GO:0006839	mitochondrial transport	74	0.29804105	3.0800812	0	4.79E-06	0.001	8534	tags=55%, list=26%, signal=74%
GO:0051640	organelle localization	201	0.18706006	3.0700545	0	4.76E-06	0.001	8540	tags=44%, list=26%, signal=59%
GO:0006261	DNA-dependent DNA replication	51	0.36920577	3.0610275	0	4.73E-06	0.001	8605	tags=63%, list=26%, signal=85%
GO:0071824	protein-DNA complex subunit organization	51	0.36908528	3.0252757	0	4.68E-06	0.001	8609	tags=63%, list=26%, signal=85%
GO:0032844	regulation of homeostatic process	282	0.15523265	2.9721014	0	4.60E-06	0.001	10092	tags=46%, list=30%, signal=65%
GO:0031396	regulation of protein ubiquitination	113	0.23220588	2.9472594	0	4.57E-06	0.001	7900	tags=47%, list=24%, signal=61%
GO:0050865	regulation of cell activation	335	0.13906783	2.932502	0	1.34E-05	0.003	10211	tags=44%, list=31%, signal=64%

GO:0070646	protein modification by small protein removal	75	0.28339273	2.9289825	0	1.77E-05	0.004	9218	tags=56%, list=28%, signal=77%
GO:0051321	meiotic cell cycle	101	0.24785416	2.9274647	0	1.76E-05	0.004	8573	tags=50%, list=26%, signal=68%
GO:0006323	DNA packaging	54	0.3365832	2.9004579	0	2.59E-05	0.006	8530	tags=59%, list=26%, signal=80%
GO:0043414	macromolecule methylation	117	0.23143785	2.886418	0	2.58E-05	0.006	8530	tags=49%, list=26%, signal=65%
GO:0010498	proteasomal protein catabolic process	110	0.23338087	2.8803222	0	3.81E-05	0.009	8588	tags=49%, list=26%, signal=66%
GO:0051302	regulation of cell division	115	0.22380655	2.871471	0	3.77E-05	0.009	8775	tags=49%, list=26%, signal=66%
GO:0006275	regulation of DNA replication	92	0.25450647	2.863415	0	3.75E-05	0.009	8547	tags=51%, list=26%, signal=69%
GO:0071478	cellular response to radiation	79	0.27556917	2.861568	0	3.73E-05	0.009	7694	tags=51%, list=23%, signal=66%
GO:0009725	response to hormone stimulus	363	0.13235202	2.846107	0	3.70E-05	0.009	10211	tags=44%, list=31%, signal=63%
GO:0001824	blastocyst development	72	0.28723127	2.843375	0	3.68E-05	0.009	8942	tags=56%, list=27%, signal=76%
GO:0071805	potassium ion transmembrane transport	121	0.22129007	2.8198617	0	3.64E-05	0.009	19324	tags=80%, list=58%, signal=191%
GO:0007517	muscle organ development	194	0.17131089	2.8195271	0	3.62E-05	0.009	10619	tags=49%, list=32%, signal=72%
GO:0015850	organic hydroxy compound transport	74	0.27273202	2.8060503	0	3.98E-05	0.01	8475	tags=53%, list=25%, signal=71%
GO:0030198	extracellular matrix organization	143	0.19884458	2.763261	0	5.94E-05	0.015	8530	tags=45%, list=26%, signal=61%
GO:0048610	cellular process involved in reproduction	337	0.1302919	2.755669	0	7.08E-05	0.018	9623	tags=42%, list=29%, signal=58%
GO:0071705	nitrogen compound transport	272	0.14023651	2.7311828	0	8.10E-05	0.021	8577	tags=40%, list=26%, signal=53%
GO:0043062	extracellular structure organization	144	0.19568029	2.7120702	0	8.71E-05	0.023	8530	tags=45%, list=26%, signal=60%
GO:0006813	potassium ion transport	132	0.19992867	2.7055109	0	9.42E-05	0.025	19324	tags=78%, list=58%, signal=186%
GO:0043484	regulation of RNA splicing	59	0.2925913	2.7033465	0	9.75E-05	0.026	8322	tags=54%, list=25%, signal=72%
GO:0034599	cellular response to oxidative stress	112	0.21821173	2.7014441	0	9.71E-05	0.026	7909	tags=46%, list=24%, signal=60%
GO:0052547	regulation of peptidase activity	272	0.13863705	2.6739967	0	1.30E-04	0.035	6796	tags=34%, list=20%, signal=43%
GO:0042593	glucose homeostasis	121	0.2034039	2.667982	0	1.41E-04	0.038	9199	tags=48%, list=28%, signal=66%
GO:1901698	response to nitrogen compound	501	0.10092685	2.6494074	0	1.46E-04	0.04	8574	tags=36%, list=26%, signal=47%
GO:0055088	lipid homeostasis	77	0.254814	2.6454492	0	1.55E-04	0.042	9683	tags=55%, list=29%, signal=77%
GO:0014706	striated muscle tissue development	240	0.14914125	2.624339	0	1.91E-04	0.051	8792	tags=41%, list=26%, signal=56%
GO:0021782	glial cell development	59	0.29089195	2.6229906	0	1.90E-04	0.051	8942	tags=56%, list=27%, signal=76%
GO:0001890	placenta development	134	0.19077103	2.6223893	0	1.89E-04	0.051	8570	tags=45%, list=26%, signal=60%
GO:0051241	negative regulation of multicellular organismal process	318	0.12669249	2.6156278	0	2.06E-04	0.056	8479	tags=38%, list=25%, signal=51%
GO:0048871	multicellular organismal homeostasis	159	0.17659664	2.614055	0	2.05E-04	0.056	8585	tags=43%, list=26%, signal=58%
GO:0033500	carbohydrate homeostasis	121	0.2034039	2.610297	0	2.15E-04	0.059	9199	tags=48%, list=28%, signal=66%
GO:0050878	regulation of body fluid levels	161	0.17227031	2.5730689	0	2.63E-04	0.074	9995	tags=47%, list=30%, signal=67%
GO:0044262	cellular carbohydrate metabolic process	84	0.23767315	2.532458	0	3.55E-04	0.101	8346	tags=49%, list=25%, signal=65%
GO:0061136	regulation of proteasomal protein catabolic process	81	0.23715718	2.529238	0	3.60E-04	0.103	8553	tags=49%, list=26%, signal=66%
GO:0061458	reproductive system development	223	0.1438763	2.5220964	0	3.79E-04	0.108	8667	tags=40%, list=26%, signal=54%
GO:0048520	positive regulation of behavior	101	0.2162302	2.5184104	0	3.87E-04	0.11	7646	tags=45%, list=23%, signal=58%
GO:0044242	cellular lipid catabolic process	97	0.22352554	2.5120099	0	3.99E-04	0.114	8357	tags=47%, list=25%, signal=63%
GO:0071214	cellular response to abiotic stimulus	131	0.1853334	2.5080063	0	4.07E-04	0.117	8329	tags=44%, list=25%, signal=58%
GO:0016042	lipid catabolic process	117	0.20235017	2.5050256	0	4.12E-04	0.119	8357	tags=45%, list=25%, signal=60%
GO:0006066	alcohol metabolic process	164	0.16332719	2.4501996	0	6.05E-04	0.179	8585	tags=42%, list=26%, signal=56%
GO:0006639	acylglycerol metabolic process	57	0.27350664	2.442612	0	6.11E-04	0.183	8421	tags=53%, list=25%, signal=70%
GO:0003018	vascular process in circulatory system	77	0.23805712	2.438942	0	6.37E-04	0.19	7216	tags=45%, list=22%, signal=58%
GO:0048872	homeostasis of number of cells	169	0.1612845	2.4382162	0	6.38E-04	0.19	8633	tags=42%, list=26%, signal=56%
GO:0016050	vesicle organization	88	0.22332267	2.4247236	0	7.01E-04	0.21	8463	tags=48%, list=25%, signal=64%
GO:0045637	regulation of myeloid cell differentiation	139	0.17408213	2.4218872	0	6.99E-04	0.211	8588	tags=43%, list=26%, signal=58%
GO:0006006	glucose metabolic process	75	0.23719203	2.414232	0	7.23E-04	0.218	8534	tags=49%, list=26%, signal=66%
GO:0007548	sex differentiation	204	0.1470891	2.412705	0	7.24E-04	0.219	8667	tags=41%, list=26%, signal=55%
GO:0007610	behavior	462	0.09530542	2.3805897	0	8.61E-04	0.257	10549	tags=41%, list=32%, signal=59%
GO:0051604	protein maturation	160	0.16269946	2.3752468	0	8.93E-04	0.267	9994	tags=46%, list=30%, signal=66%
GO:0044744	protein targeting to nucleus	74	0.23053376	2.3656347	0.002079	9.59E-04	0.286	8527	tags=49%, list=26%, signal=65%

Supplementary Table 8: Gene Set Enrichment Analysis for highly enriched genes in si14-3-3ζ-transfected 3T3-L1 cells at t=48 hr of differentiation

Term ID	Description	SIZE	ES	NES	NOM p-val	FDR q-val	FWER p-val	RANK AT MAX	LEADING EDGE
GO:0016192	vesicle-mediated transport	489	-0.2361505	-6.080085	0	0	0	8720	tags=49%, list=26%, signal=66%
GO:0051338	regulation of transferase activity	502	-0.2227057	-5.769673	0	0	0	8671	tags=48%, list=26%, signal=64%
GO:0030029	actin filament-based process	273	-0.2970658	-5.734875	0	0	0	8718	tags=56%, list=26%, signal=75%
GO:0051726	regulation of cell cycle	527	-0.2115478	-5.602441	0	0	0	8663	tags=47%, list=26%, signal=62%
GO:0040012	regulation of locomotion	497	-0.2185218	-5.5083847	0	0	0	8633	tags=47%, list=26%, signal=63%
GO:0044087	regulation of cellular component biogenesis	355	-0.231789	-5.1717477	0	0	0	8674	tags=49%, list=26%, signal=66%
GO:0009118	regulation of nucleoside metabolic process	322	-0.2468043	-5.038128	0	0	0	8603	tags=50%, list=26%, signal=67%
GO:0016265	death	558	-0.1833818	-4.9549775	0	0	0	8665	tags=44%, list=26%, signal=59%
GO:0043009	chordate embryonic development	565	-0.1818715	-4.8722143	0	0	0	8946	tags=45%, list=27%, signal=60%
GO:0051674	localization of cell	578	-0.1724396	-4.7479477	0	0	0	8634	tags=43%, list=26%, signal=57%
GO:0044782	cilium organization	118	-0.3615969	-4.6650243	0	0	0	8591	tags=62%, list=26%, signal=83%
GO:0035295	tube development	417	-0.1997826	-4.6648045	0	0	0	7954	tags=44%, list=24%, signal=57%
GO:0001934	positive regulation of protein phosphorylation	497	-0.18468	-4.659271	0	0	0	8671	tags=44%, list=26%, signal=59%
GO:0051960	regulation of nervous system development	543	-0.1740141	-4.6578426	0	0	0	8700	tags=43%, list=26%, signal=58%
GO:0022610	biological adhesion	360	-0.2121174	-4.626606	0	0	0	8634	tags=47%, list=26%, signal=63%
GO:0061024	membrane organization	354	-0.2132091	-4.554719	0	0	0	8016	tags=45%, list=24%, signal=59%
GO:0006508	proteolysis	341	-0.2123188	-4.5170197	0	0	0	8713	tags=47%, list=26%, signal=63%
GO:0001501	skeletal system development	338	-0.2123708	-4.515681	0	0	0	8850	tags=48%, list=27%, signal=64%
GO:0007155	cell adhesion	353	-0.2043017	-4.414575	0	0	0	8634	tags=46%, list=26%, signal=62%
GO:0007017	microtubule-based process	310	-0.2080735	-4.350895	0	0	0	8700	tags=47%, list=26%, signal=63%
GO:0040008	regulation of growth	461	-0.1722964	-4.171805	0	0	0	8633	tags=43%, list=26%, signal=57%
GO:0007167	enzyme linked receptor protein signaling pathway	372	-0.1899223	-4.163928	0	0	0	8684	tags=45%, list=26%, signal=60%
GO:0016197	endosomal transport	101	-0.3430935	-4.123549	0	0	0	8710	tags=60%, list=26%, signal=82%
GO:0060627	regulation of vesicle-mediated transport	247	-0.2212148	-4.033931	0	0	0	8720	tags=48%, list=26%, signal=65%
GO:0051052	regulation of DNA metabolic process	226	-0.2233231	-4.0264244	0	0	0	8663	tags=48%, list=26%, signal=65%
GO:0043069	negative regulation of programmed cell death	540	-0.1510924	-3.964681	0	0	0	8544	tags=41%, list=26%, signal=54%
GO:0007018	microtubule-based movement	106	-0.3364804	-3.9404993	0	0	0	8611	tags=59%, list=26%, signal=80%
GO:0007033	vacuole organization	51	-0.4419144	-3.7341652	0	0	0	8149	tags=69%, list=25%, signal=91%
GO:0001822	kidney development	154	-0.2531526	-3.7174761	0	0	0	8680	tags=51%, list=26%, signal=69%
GO:0009607	response to biotic stimulus	419	-0.1559732	-3.7136965	0	0	0	8768	tags=42%, list=26%, signal=56%
GO:0001655	urogenital system development	222	-0.2193321	-3.6954725	0	0	0	6237	tags=41%, list=19%, signal=50%
GO:0006914	autophagy	50	-0.4356172	-3.6658747	0	0	0	8149	tags=68%, list=25%, signal=90%
GO:0007389	pattern specification process	373	-0.1595423	-3.6485147	0	0	0	9019	tags=43%, list=27%, signal=58%
GO:0043207	response to external biotic stimulus	407	-0.1559459	-3.641813	0	0	0	8768	tags=42%, list=26%, signal=56%
GO:0048736	appendage development	144	-0.2459476	-3.5145888	0	0	0	8946	tags=51%, list=27%, signal=70%
GO:0034330	cell junction organization	99	-0.2973587	-3.4823043	0	0	0	8616	tags=56%, list=26%, signal=75%
GO:0090066	regulation of anatomical structure size	220	-0.1897631	-3.3558002	0	0	0	8545	tags=45%, list=26%, signal=60%
GO:0019953	sexual reproduction	447	-0.1385429	-3.3028207	0	0	0	8623	tags=40%, list=26%, signal=53%
GO:0003002	regionalization	272	-0.1743102	-3.2917876	0	0	0	9289	tags=45%, list=28%, signal=62%
GO:0005975	carbohydrate metabolic process	291	-0.1645853	-3.2805629	0	0	0	8174	tags=41%, list=25%, signal=54%
GO:0006650	glycerophospholipid metabolic process	124	-0.2504226	-3.271072	0	0	0	8331	tags=50%, list=25%, signal=66%
GO:0032970	regulation of actin filament-based process	199	-0.1993976	-3.2358994	0	0	0	8616	tags=46%, list=26%, signal=61%
GO:0030855	epithelial cell differentiation	347	-0.1482942	-3.1943257	0	0	0	8633	tags=41%, list=26%, signal=54%
GO:0009611	response to wounding	353	-0.1447205	-3.1915536	0	0	0	8616	tags=40%, list=26%, signal=54%
GO:0006366	transcription from RNA polymerase II promoter	381	-0.1405681	-3.1911757	0	0	0	8559	tags=40%, list=26%, signal=53%
GO:0050776	regulation of immune response	342	-0.144324	-3.116725	0	0	0	8766	tags=41%, list=26%, signal=55%
GO:0007224	smoothened signaling pathway	60	-0.3347433	-3.0625434	0	0	0	7733	tags=57%, list=23%, signal=74%
GO:0001503	ossification	142	-0.2161579	-3.0522537	0	0	0	8065	tags=46%, list=24%, signal=60%
GO:0071396	cellular response to lipid	195	-0.1862987	-3.0480232	0	0	0	15840	tags=66%, list=48%, signal=126%
GO:0050678	regulation of epithelial cell proliferation	221	-0.1739182	-3.0351386	0	0	0	9152	tags=45%, list=28%, signal=61%
GO:0009636	response to toxic substance	79	-0.2923874	-3.0161343	0	3.69E-06	0.001	7559	tags=52%, list=23%, signal=67%
GO:0071495	cellular response to endogenous stimulus	510	-0.1144523	-2.990661	0	3.63E-06	0.001	8446	tags=37%, list=25%, signal=48%
GO:0000910	cytokinesis	62	-0.3168415	-2.9571779	0	7.00E-06	0.002	7720	tags=55%, list=23%, signal=71%

Supplementary Table 9: RNA-Seq analysis of significantly changed genes between WT and 14-3-3ζKO WAT

	Gene Name	WT	KO	log2_fold_change	test_stat	p_value	q_value	significant
**1**	Gsta3	60.1482	220.266	1.87266	1.6886	0.00005	0.0168529	yes
**2**	Cd84	74.7583	10.8118	-2.78962	-1.87853	0.00005	0.0168529	yes
**3**	Zranb3	11.7006	1.76169	-2.73155	-1.57406	0.0001	0.0291356	yes
**4**	Ptprc	28.3934	7.61966	-1.89776	-1.62167	0.0001	0.0291356	yes
**5**	Soat1	18.3116	4.54622	-2.01002	-1.80544	0.0001	0.0291356	yes
**6**	Fcgr3	132.566	31.2613	-2.08426	-1.88792	0.00005	0.0168529	yes
**7**	Itgb2	95.1603	15.2225	-2.64415	-2.00548	0.0002	0.0419268	yes
**8**	Lum	63.7533	16.9959	-1.90731	-1.86223	0.00015	0.0353219	yes
**9**	Gas2l3	3.6384	0.445201	-3.03077	-1.81063	0.00015	0.0353219	yes
**10**	Col1a1	60.0326	10.2653	-2.54797	-2.12031	0.00005	0.0168529	yes
**11**	Cd68	272.485	42.7278	-2.67293	-2.11219	0.00005	0.0168529	yes
**12**	Slc4a1	0.227586	4.39131	4.27017	1.19142	0.00005	0.0168529	yes
**13**	Cd300lb	29.8549	2.86461	-3.38155	-2.19327	0.00005	0.0168529	yes
**14**	Cd300ld	26.497	4.22587	-2.64851	-2.18922	0.00005	0.0168529	yes
**15**	AF251705,Gm11709	43.3304	5.98087	-2.85695	-2.30166	0.00005	0.0168529	yes
**16**	Ahnak2	10.419	1.73855	-2.58326	-1.99768	0.00005	0.0168529	yes
**17**	Ighg3	3.12384	77.7305	4.63709	2.71027	0.00005	0.0168529	yes
**18**	Gadd45g	23.729	115.464	2.28271	1.86996	0.0002	0.0419268	yes
**19**	Sema4d	8.40211	1.97682	-2.08757	-1.48663	0.00005	0.0168529	yes
**20**	Cma1	38.7107	5.70688	-2.76196	-2.03026	0.00015	0.0353219	yes
**21**	Ywhaz	107.232	22.5113	-2.25201	-2.05306	0.0002	0.0419268	yes
**22**	Nfam1	16.9394	2.69452	-2.65229	-1.88883	0.00015	0.0353219	yes
**23**	Cd200r1	15.3232	1.69513	-3.17625	-1.8142	0.0002	0.0419268	yes
**24**	Tpsb2	33.404	4.79389	-2.80075	-1.75352	0.0002	0.0419268	yes
**25**	Myo1f	35.8196	3.74727	-3.25684	-2.02719	0.00015	0.0353219	yes
**26**	Ier3	17.0311	84.8353	2.31649	2.23432	0.00005	0.0168529	yes
**27**	Trem2	182.24	15.2947	-3.57474	-2.58457	0.00005	0.0168529	yes
**28**	Emr1	95.5065	7.78994	-3.61592	-2.65646	0.00005	0.0168529	yes
**29**	Mpeg1	142.319	17.8949	-2.99151	-2.13241	0.00005	0.0168529	yes
**30**	Dock8	15.2778	4.58114	-1.73766	-1.57149	0.00025	0.0499709	yes
**31**	Ms4a7	63.9103	7.61632	-3.06888	-2.10948	0.00005	0.0168529	yes
**32**	Lipa	158.049	25.7894	-2.61552	-2.0193	0.0002	0.0419268	yes
**33**	Pik3ap1	16.4405	2.40731	-2.77176	-2.20135	0.00005	0.0168529	yes
**34**	Arhgap19	12.3071	2.11964	-2.5376	-1.70941	0.00015	0.0353219	yes
**35**	Il1rn	16.0331	1.63234	-3.29604	-1.63609	0.00015	0.0353219	yes
**36**	AA467197,Mir147	3.02019	31.8232	3.39737	1.62029	0.00005	0.0168529	yes
**37**	SNORA71,Snhg11	10.8806	45.5648	2.06616	1.75754	0.00005	0.0168529	yes
**38**	Cd44	36.8633	9.7028	-1.92571	-1.64932	0.00015	0.0353219	yes
**39**	Atp8b4	4.09551	0.588127	-2.79984	-1.71761	0.00005	0.0168529	yes
**40**	Siglec1	7.49662	1.57024	-2.25526	-1.80861	0.0002	0.0419268	yes
**41**	Mafb	48.1098	11.886	-2.01707	-1.88804	0.00015	0.0353219	yes
**42**	Nceh1	23.0693	4.18831	-2.46153	-2.04581	0.00005	0.0168529	yes
**43**	Ctsk	111.615	19.9449	-2.48444	-2.25629	0.00005	0.0168529	yes
**44**	Ctss	572.263	86.434	-2.72701	-2.16688	0.0001	0.0291356	yes
**45**	Sdc3	39.9913	10.4489	-1.93633	-1.76606	0.0002	0.0419268	yes
**46**	Ptafr	24.3889	2.79531	-3.12514	-2.57622	0.00005	0.0168529	yes
**47**	Atp6v0d2	93.7766	12.4209	-2.91646	-2.34642	0.00005	0.0168529	yes
**48**	C1qb	359.144	92.2969	-2.32921	-1.5227	0.0002	0.0419268	yes
**49**	Acacb	6.93223	59.5802	3.10344	1.94199	0.00005	0.0168529	yes
**50**	Daglb	27.7535	6.65146	-2.06093	-1.72523	0.00005	0.0168529	yes
**51**	Capg	264.099	62.2081	-2.0859	-1.85883	0.0001	0.0291356	yes
**52**	Clec4a3	29.5356	2.82491	-3.38618	-2.07489	0.00005	0.0168529	yes
**53**	Clec4a2	46.0799	6.71327	-2.77905	-1.87882	0.00005	0.0168529	yes
**54**	C530028O21Rik	11.8474	1.58346	-2.90343	-1.76085	0.00005	0.0168529	yes
**55**	Clec12a	49.7811	4.11072	-3.59813	-2.54298	0.00005	0.0168529	yes
**56**	Apold1	4.06442	25.3431	2.64047	1.74406	0.00005	0.0168529	yes
**57**	Arhgap25	35.7297	6.05842	-2.56011	-1.94177	0.00005	0.0168529	yes
**58**	Apobec1	76.816	14.1357	-2.44206	-1.80742	0.00015	0.0353219	yes
**59**	C3ar1	38.1332	5.25539	-2.85918	-2.4003	0.00005	0.0168529	yes
**60**	Clec7a	49.6681	8.5276	-2.54211	-1.92213	0.0001	0.0291356	yes
**61**	2310014L17Rik,U6	0.488944	139.327	8.15459	3.07234	0.00005	0.0168529	yes
**62**	Cyp2f2	2.08	45.5433	4.45258	3.14411	0.00005	0.0168529	yes
**63**	Tyrobp	469.755	71.8516	-2.70881	-3.21639	0.00005	0.0168529	yes
**64**	Itgax	49.0664	5.98807	-3.03457	-1.99853	0.00005	0.0168529	yes
**65**	Gm14548,Gm15922,Lilrb3	33.718	6.97633	-2.27298	-1.707	0.00005	0.0168529	yes
**66**	Lair1	15.3399	1.4875	-3.36634	-1.51053	0.00015	0.0353219	yes
**67**	Atp1a3	17.409	2.63824	-2.72219	-1.74069	0.00005	0.0168529	yes
**68**	Anpep	55.4056	7.40268	-2.90391	-2.32152	0.00005	0.0168529	yes
**69**	Adam8	64.7107	8.51135	-2.92654	-1.61393	0.00005	0.0168529	yes
**70**	Msr1	22.8286	3.10179	-2.87967	-1.7845	0.00005	0.0168529	yes
**71**	Ces1f	26.447	131.871	2.31795	1.99936	0.00005	0.0168529	yes
**72**	Cotl1	131.618	30.6453	-2.10261	-1.86318	0.00015	0.0353219	yes
**73**	Mmp7	15.744	126.278	3.00373	2.05297	0.00025	0.0499709	yes
**74**	S1pr2	10.712	1.55295	-2.78615	-2.00295	0.00025	0.0499709	yes
**75**	Slc37a2	30.1536	2.13361	-3.82096	-2.4929	0.00005	0.0168529	yes
**76**	Tlr13	16.2404	2.4535	-2.72668	-1.98869	0.00005	0.0168529	yes
**77**	Cybb	36.7509	5.6771	-2.69455	-2.24621	0.00005	0.0168529	yes
**78**	Tlr8	13.2891	1.06259	-3.64458	-2.28404	0.00005	0.0168529	yes
